# NAD^+^ bioavailability mediates PARG inhibition-induced replication arrest, intra S-phase checkpoint and apoptosis in glioma stem cells

**DOI:** 10.1093/narcan/zcab044

**Published:** 2021-11-17

**Authors:** Jianfeng Li, Kate M. Saville, Md Ibrahim, Xuemei Zeng, Steve McClellan, Anusha Angajala, Alison Beiser, Joel F Andrews, Mai Sun, Christopher A Koczor, Jennifer Clark, Faisal Hayat, Mikhail V Makarov, Anna Wilk, Nathan A Yates, Marie E Migaud, Robert W Sobol

**Affiliations:** Mitchell Cancer Institute, University of South Alabama, Mobile, AL 36604, USA; Department of Pharmacology, College of Medicine, University of South Alabama, Mobile, AL 36604, USA; Mitchell Cancer Institute, University of South Alabama, Mobile, AL 36604, USA; Department of Pharmacology, College of Medicine, University of South Alabama, Mobile, AL 36604, USA; Mitchell Cancer Institute, University of South Alabama, Mobile, AL 36604, USA; Department of Pharmacology, College of Medicine, University of South Alabama, Mobile, AL 36604, USA; Biomedical Mass Spectrometry Center, University of Pittsburgh Schools of the Health Sciences, Pittsburgh, PA 15213, USA; Mitchell Cancer Institute, University of South Alabama, Mobile, AL 36604, USA; Mitchell Cancer Institute, University of South Alabama, Mobile, AL 36604, USA; Department of Pharmacology, College of Medicine, University of South Alabama, Mobile, AL 36604, USA; Mitchell Cancer Institute, University of South Alabama, Mobile, AL 36604, USA; Department of Pharmacology, College of Medicine, University of South Alabama, Mobile, AL 36604, USA; Mitchell Cancer Institute, University of South Alabama, Mobile, AL 36604, USA; Biomedical Mass Spectrometry Center, University of Pittsburgh Schools of the Health Sciences, Pittsburgh, PA 15213, USA; Mitchell Cancer Institute, University of South Alabama, Mobile, AL 36604, USA; Department of Pharmacology, College of Medicine, University of South Alabama, Mobile, AL 36604, USA; Mitchell Cancer Institute, University of South Alabama, Mobile, AL 36604, USA; Department of Pharmacology, College of Medicine, University of South Alabama, Mobile, AL 36604, USA; Mitchell Cancer Institute, University of South Alabama, Mobile, AL 36604, USA; Department of Pharmacology, College of Medicine, University of South Alabama, Mobile, AL 36604, USA; Mitchell Cancer Institute, University of South Alabama, Mobile, AL 36604, USA; Department of Pharmacology, College of Medicine, University of South Alabama, Mobile, AL 36604, USA; Mitchell Cancer Institute, University of South Alabama, Mobile, AL 36604, USA; Department of Pharmacology, College of Medicine, University of South Alabama, Mobile, AL 36604, USA; Biomedical Mass Spectrometry Center, University of Pittsburgh Schools of the Health Sciences, Pittsburgh, PA 15213, USA; Department of Cell Biology, University of Pittsburgh School of Medicine, Pittsburgh, PA 15261, USA; Mitchell Cancer Institute, University of South Alabama, Mobile, AL 36604, USA; Department of Pharmacology, College of Medicine, University of South Alabama, Mobile, AL 36604, USA; Mitchell Cancer Institute, University of South Alabama, Mobile, AL 36604, USA; Department of Pharmacology, College of Medicine, University of South Alabama, Mobile, AL 36604, USA

## Abstract

Elevated expression of the DNA damage response proteins PARP1 and poly(ADP-ribose) glycohydrolase (PARG) in glioma stem cells (GSCs) suggests that glioma may be a unique target for PARG inhibitors (PARGi). While PARGi-induced cell death is achieved when combined with ionizing radiation, as a single agent PARG inhibitors appear to be mostly cytostatic. Supplementation with the NAD^+^ precursor dihydronicotinamide riboside (NRH) rapidly increased NAD^+^ levels in GSCs and glioma cells, inducing PARP1 activation and mild suppression of replication fork progression. Administration of NRH+PARGi triggers hyperaccumulation of poly(ADP-ribose) (PAR), intra S-phase arrest and apoptosis in GSCs but minimal PAR induction or cytotoxicity in normal astrocytes. PAR accumulation is regulated by select PARP1- and PAR-interacting proteins. The involvement of XRCC1 highlights the base excision repair pathway in responding to replication stress while enhanced interaction of PARP1 with PCNA, RPA and ORC2 upon PAR accumulation implicates replication associated PARP1 activation and assembly with pre-replication complex proteins upon initiation of replication arrest, the intra S-phase checkpoint and the onset of apoptosis.

## INTRODUCTION

Glioma stem cells (GSCs) are capable of extensive self-renewal and multi-lineage differentiation and therefore paramount in the initiation and development of glioma (1,2). These pro-growth phenotypes promote therapeutic resistance and recurrence of glioblastoma multiforme (GBM) (3–5). Glioma tumors, glioma-derived cells and particularly GSCs are thought to be resistant to DNA damage treatment due to increased expression of the DNA repair machinery (6–9) and of select DNA damage response (DDR) genes (10,11), including PARP1, a critical DNA damage sensor and DNA repair coordinator (12). Damaged DNA rapidly promotes PARP1-mediated poly-(ADP-ribose) modification (PARylation) of itself and other target proteins to facilitate chromatin-relaxation and the recruitment of DNA repair and DDR factors to the site of the DNA lesion (13). Upon completion of repair, the timely degradation of PAR (dePARylation) by ADP-ribose glycohydrolases such as PARG and ARH3 promotes DNA repair complex disassembly (14) and completion of the repair process. Many tumor cells express elevated levels of PARP1 and PARG, such as GSCs as we describe herein. However, NAD^+^ is an essential substrate for PARP1-mediated PAR synthesis and is therefore a limiting factor for PARP1 activity potential (15–17). Specifically, decreased levels of cellular NAD^+^ reduce cellular PARP1 activation (17), leading to a defect in cellular DNA repair capacity (15) and suppression of DNA repair protein complex formation (14,15) while increasing NAD^+^ bioavailability enhances DNA repair protein complex assembly (14). Given the variability of cellular levels of NAD^+^ across different organs and the numerous mechanisms of NAD^+^ biosynthetic pathways that are altered in cancer (18,19), clarifying the essential role of NAD^+^ in PARP1 activation and the cellular response to DNA damage is essential (16).

In addition to PARP1’s critical role as a key signal transduction protein in response to exogenously induced DNA damage (e.g. global base excision repair and single-strand break repair, BER/SSBR), recent efforts have uncovered a role for PARP1 in responding to replication stress (20). As early as 1998, PARP1 was suggested to be a part of the ‘DNA synthesome’ in transformed cells, although to what extent was not clear (21). PARP1 has since been suggested to have several possible roles in response to replication stress. Regarding replication fork remodeling and processing, PARP1 interacts with and signals to RECQ1 to suppress replication restart (22). Further, PARP1 is associated with recognition of Okazaki fragments and recruits XRCC1 (23–25) to aid BRCA2 to block fork degradation by MRE11 (26,27). Further, it has been suggested that PARP1-induced PARylation facilitates the intra S-phase checkpoint via CHK1 activation (28). Finally, PARP1-mediated PARylation acts to throttle replication fork speed and conversely, PARP1 inhibition (PARPi) enhances replication fork progression, further elevating replication stress (29). This unrestrained fork progression by PARP1 inhibition therefore promotes the synthetic lethality observed in homologous recombination deficient cells and cancers (30).

Acquired resistance to PARPi has been described in pre-clinical and clinical studies, which suggests the need to identify other protein targets (31). DNA damage-induced or replication-associated PARP1 activation requires the ADP-ribose glycohydrolase activity of PARG to prevent PAR-induced cell death (13,17,28), implicating PARG as a potentially novel and effective target. Potent PARG inhibitors (PARGis) have been developed (PDD00017273 and JA2131) (32,33) that show promise when combined with irradiation (34) but are minimally effective (against ovarian cancer cells) as a single agent unless targeted to cancers with replication defects or combined with CHK1 inhibitors (35). With the significantly elevated PARP1 and PARG protein levels seen in glioma-derived cells and GSCs (herein), we reasoned that PARGis may be effective to enhance PAR-induced cell death following DNA damage-mediated PARP1 activation (e.g. following irradiation) and from replication-associated PARP1 activation. As with earlier reports, we find that PARGi-induced cell death and cell cycle arrest at G2/M in GSCs is dependent on induced DNA damage and PARP1 activation, such as that from irradiation, but as a single agent is primarily cytostatic and does not induce cell death. This suggests that replication associated PARP1 activation may be suppressed in glioma. Given the requirement of NAD^+^ for PARP1 activation, we reasoned that constrained or insufficient cellular levels of NAD^+^ would limit PARP1 activation mediated suppression of replication fork progression. In-line with this hypothesis, supplementation with the NAD^+^ precursor dihydronicotinamide riboside (NRH) rapidly increased cellular NAD^+^ levels, inducing PARP1 activation and mild suppression of replication fork progression. Subsequently, the simultaneous administration of a PARG inhibitor with NRH triggers accumulation of PAR mediated by replication-dependent PARP1 activation, induction of an intra S-phase cell cycle arrest and near 100% apoptosis-mediated cell death. Further, PAR accumulation is regulated by and promotes assembly of select PARP1 and PAR interacting proteins such as XRCC1, PCNA and ORC2. The involvement of XRCC1 highlights the role of the BER/SSBR pathway in responding to replication stress while the enhanced interaction of PARP1 with PCNA, RPA and ORC2 upon PAR accumulation suggests that replication associated PARP1 activation and assembly with pre-replication complex proteins initiates replication arrest and apoptosis.

## MATERIALS AND METHODS

### Cells and cell culture conditions

Glioma stem cells (GSCs) derived from high-grade glioma (HGG) samples, either the MES subtype (GSC-83, GSC-326) or the PN subtype (GSC-19, GSC-84), were described previously (11). All cells were cultured at 37°C with 5% CO_2_. Details for each cell line used herein are detailed in Supplementary Table S1 and detailed protocols are found in the Supplementary Methods. Cells were treated with the following inhibitors, as described below and in the legends: PARP1/2 inhibitor (ABT-888, Veliparib), Tocris, Cat. No. 7026; PARG inhibitor (PDD00017273), Tocris, Cat. No. 5952/1 and Sigma-Aldrich, Cat. No. SML1781 and FEN1 inhibitor (LNT 1), Tocris, Cat. No. 6510.

### Quantitative real-time PCR (qRT-PCR)

The expression of PARP1 mRNA and PARG mRNA was determined using Taqman Gene Expression Assay probes from Life Technologies (PARP1: probe ID: Hs00242302_m1; PARG: probe Hs00608254_m1). β-Actin (probe ID: Hs99999903_m1) was used as an internal control. The qRT-PCR reactions were performed in an ABI StepOnePlus RT-PCR system according to the manufacturer’s protocol. Analysis of mRNA expression was performed as per the instruction of the manufacturer (ΔΔCT method). Samples were run in triplicate and the results shown are the mean ±SD of three analyses.

### Cell extract for immunoblot analysis

Cells (5 × 10^5^) were seeded in a 60 mm dish and treated in 5 ml growth medium. Then, cells were collected and were lysed using 2× clear Laemmli buffer at a ratio of 100 μl Laemmli buffer per 10^6^ cells. Whole cell lysate (15 μl, ∼30 μg) was used for immunoblot analyses. The primary antibodies used are listed in Supplementary Table S2.

### Lentivirus production and cell transduction

Lentiviral particles were generated by co-transfection of four plasmids into 293-FT cells using the TransIT-X2 Transfection reagent, including the packaging vectors (pMD2.g(VSVG), pVSV-REV and pMDLg/pRRE) and the Cas9 and gRNA expressing shuttle vectors designed to target PARP1 or XRCC1 (kindly provided by Wim Vermeulen, Erasmus MC, Netherlands). Lentivirus-containing supernatant was collected 48 h after transfection and then passed through 0.45 mM filters to isolate the viral particles, as described previously (13,36). The lentivirus particles were then further concentrated using Lenti-X Concentrator (Takara Bio, Cat# 631231), as per the manufacturer’s instructions.

For lentiviral transduction, Glioma stem cells (GSC-83) (1–2 × 10^5^) were seeded into six-well plates with polybrene containing growth medium (1 ml, final polybrene concentration is 8 μg/ml). Next, the concentrated lentiviral particles were immediately added into the well and then mixed with the cells. Cells were incubated at 32°C overnight, and then medium with lentiviral particles was removed and replaced with fresh medium. The cells were then cultured at 37°C for up to 2 weeks for subsequent analyses.

### PARP1 and XRCC1 knockout by CRISPR/Cas9

Guide RNAs (gRNAs) targeting PARP1 or XRCC1 were designed using the CRISPR Design Tool (37), and as described (38) (kindly provided by Wim Vermeulen, Erasmus MC, Netherlands). Each separate gRNA was cloned into pLentiCRISPRv2 (39). Details for each vector developed or used herein are described in Supplementary Table S3. PARP1 or XRCC1 knockout was performed as described (39,40) and confirmed by immunoblot analysis of whole cell lysates. The gRNA target sequences are listed in Supplementary Table S3. The primary and secondary antibodies antibodies used are listed in Supplementary Table S2.

### ORC2 knockout by CRISPR/Cas9 in LN428 cells

LN428/ORC2-KO cells were created by transfection of ribonucleoprotein complexes including Cas9 and a mixture of three single-guide RNAs (sgRNAs) (41) targeting an early exon of the ORC2 gene (Synthego). LN428 cells were seeded at a density of 2 × 10^5^ cells per well (six-well plate). After 24 h incubation, the cells were transfected with a mixture of sgRNAs, Cas9 and the CRISPRMAX-Cas9 transfection reagent (Cat# CMAX00008, Thermo Fisher Scientific) in serum-free OptiMEM (Cat# 31985070, Thermo Fisher Scientific). After 48 h, media containing the transfection reagent were replaced with fresh media (Media #2, Supplementary Table S1) and allowed to grow for another 2 days. Validation of gene targeting (knockout, KO) was then confirmed by immunoblot using whole cell lysates, as compared to a non-targeted control. The primary and secondary antibodies used are listed in Supplementary Table S2.

### Cell viability analysis

#### For cell viability analysis following IR treatment

GSCs (10^5^) were seeded in 60 mm dishes with 5 ml growth medium supplemented with DMSO (Control), PARPi (ABT888, 10 μM), PARGi (PDD00017273, 10 μM) or PARPi+PARGi for 30 min and then irradiated using an X-Rad 320 system (Precision X-Ray, North Branford, CT). Next, the treated cells were incubated for 5 days. The cells were then collected and counted by Trypan Blue exclusion.

#### For cell viability analysis following NRH, PARGi or NRH±PARGi treatment

GSCs (10^5^) were seeded in 60 mm dishes with 5 ml growth medium supplemented with DMSO (Control), NRH (100 μM), PARGi (doses as at indicated in the figure legends) or NRH+PARGi and incubated for 5 days. The cells were then collected and counted by Trypan Blue exclusion. For the LN428 or astrocyte cells, 800 cells were seeded in each well of a 96-well-plate with 100 μl growth medium and cultured overnight. On the second day, 100 μl of 2× treatment medium was added to each well, and the cells were cultured for 5 days and then the cell number of each well was counted using the Celigo Image Cytometer (Nexcelom Bioscience). Each assay is run in triplicate.

### CometChip analysis

After each treatment, GSC cells (10^5^) were loaded into each well of the CometChip for the DNA damage assay as we have described previously (42). For PARGi combined with IR or MMS, GSC-83 cells (1 × 10^6^) were seeded in a 35 mm dish with 2 ml medium supplemented with DMSO or PARGi for 30 min. The dishes were then irradiated using the X-Rad 320 system at the indicated dose or treated with MMS for 30 min. A cell suspension (100 μl) was then loaded into each well of the 30-micron CometChip for analysis. DNA damage is represented as % Tail DNA. Detailed protocols are found in the Supplementary Methods.

### NAD^+^ measurements

The NAD^+^ levels in extracts of each cell line was measured using the Enzychrome NAD^+^/NADH colorimetric assay kit (BioAssay Systems, E2ND-100) as we have described previously (16). Detailed protocols are found in the Supplementary Methods.

### Cellular ATP measurements

Cellular ATP levels were measured using the ATP lite assay (Perkin-Elmer) as per the manufacturer’s instructions. Briefly, cells (0.5 × 10^6^) were treated by DMSO or the PARG inhibitor PDD00017273 for 30 min and then irradiated using the X-Rad 320 system. After irradiation (30, 60 or 120 min), the cells were collected and then seeded (5 × 10^4^) in a 96-well plate in triplicate, and ATP content was measured by luminescence using the ATPlite assay (Perkin-Elmer). Results were shown as the fold increase of the treated cells normalized to DMSO-treated cells.

### Cell cycle analysis

Post-treatment (24 h), cells were collected, washed and then fixed with 70% ethanol at −30°C overnight. The cells were then washed twice with ice-cold PBS and resuspended in 0.5 ml FxCycle™ PI/RNase Staining Solution (cat. #F10797, Thermo Fisher Scientific). After incubation at room temperature for 30 min, DNA content of the stained cells was analyzed by flow cytometry using a FACS Canto II running Diva Software Version 8.3 (BD Biosciences San Jose, CA). The histogram of the cell cycle distribution was generated from 10 000 events per sample. The data are presented as the percentage of the cells in the G0/G1, S and G2/M phases using ModFit LT 5.0 software (Verity Software House, Topsham, ME).

### Immunoprecipitation (IP) with the PAR-binding domain resin

GSC-83 cells were treated with thymidine (2 mM) for 48 h. Half of the cells were further cultured in growth medium with thymidine (2 mM) for 1 h and then the media were supplemented with NRH (100 μM)/PARGi (10 μM) for additional 1 h. The cells were then collected for PAR analysis by immunoblot or for PAR-IP (PAR-IP, control). The other half of the cells were washed once with PBS (10 ml) and cultured in normal growth medium for 1 h followed by a treatment with NRH (100 μM)/PARGi (10 μM) for an additional 1 h (PAR-IP, 1 h Thymidine release). For each, PAR-modified or PAR-bound proteins were then either evaluated by immunoblot or captured using the PAR-agarose resin (Tulip Biolabs, Cat. #4306) as per the manufacturer’s instructions.

### EdU incorporation

GSC-83 cells were treated with DMSO, NRH (100 μM), PARGi (10 μM) or NRH (100 μM)/PARGi (10 μM) for 24 h. Then, EdU (final concentration, 10 μM) was added to the cells, except those labeled as ‘No EdU control’ and were then incubated at 37°C for an additional 2 h. Cells were collected and fixed for 15 min with fixing solution and permeabilized for 30 min at room temperature in the dark using permeabilization solution as per the manufacturer’s instructions (Thermofisher, Cat# C10635). The fixed cells were then stained with the Click‐iT™ reaction mixture and propidium iodide (PI) with RNase according to the instructions in the assay kit. Immediately after staining, the EdU incorporation was analyzed by flow cytometry using a FACS Canto II.

### DNA fiber assay

DNA replication fork progression was determined by the Replication Combing Assay (Genomic Vision, EXT-001). LN428 cells were grown to 30% confluency in 100 mm dishes and treated with DMSO, NRH (100 μM), PARGi (10 μM) or NRH+PARGi for 4 h. Following the treatment, cells were labeled with IdU (25 μM) for 30 min. Cells were washed two times with 1× PBS, trypsinized and centrifuged at 800 × *g* for 5 min. Cells were washed with 1× PBS twice, and the pellet was resuspended in an agarose plug followed by cell lysis and agarose gel digestion in disposable DNA reservoirs (Genomic Vision, RES-001). Stretching the naked DNA on coverslips (Genomic Vision, RES-001) was performed as described by the manufacturer. Coverslips were dehydrated at 65°C for 2 h followed by immunostaining. Coverslips were blocked for 30 min with Block Aid, followed by staining with mouse anti-BrdU (40 μl) and rat anti-BrdU (8 μl) in 1632 μl of Block Aid for each coverslip (1 h). Coverslips were washed with 1× PBS-Tween 0.05%, 3 times for 5 min each, on an orbital agitator plate (100 RPM) followed by addition of 2 μl of goat anti-mouse Cy3 in 1998 μl of Block Aid. Coverslips were then washed with 1× PBS-Tween 0.05%, 3 times for 5 min each, on an orbital agitator plate (100 RPM). Coverslips were mounted on a microscope slide using Prolong Gold antifade mounting media (Invitrogen, P36934), and the slides were imaged by confocal microscopy. NIS elements software length measurement tool was used to measure fiber length, graphed using Prism 8 (GraphPad Prism) and statistical significance determined by one-way Anova analysis.

### NMR analysis

NRH was dissolved in water and kept in single-use aliquots at −80ºC. After 2 months, the samples were tested for their stability. The samples were prepared for NMR analysis as follows: aqueous NRH solution (450 μl) was mixed with D_2_O (50 μl), and the resulting mixture was vortexed three times. NMR spectral acquisition (ns = 16) was then performed using a Bruker Avance III HD NMR spectrometer equipped with 400 MHz magnets Ultrashield Plus, with temperature fixed to 300 K NMR measurements. TopSpin 3.2 (Bruker BioSpin) was used for NMR spectral acquisition and preprocessing, and the automation of sample submission was performed using ICON-NMR (Bruker BioSpin). The samples were automatically shimmed.

### Statistical analysis

For most analyses, data is shown as the mean ± standard deviation from 2 to 4 independent experiments. Student’s *t*-test was used for comparisons between two groups. For multiple comparisons, one-way ANOVA was used. Statistical analysis was performed using Prism 8 (GraphPad Prism).

## RESULTS

### Upregulated PARP1 expression in glioma stem cells—impact on PARG inhibitor response

**Figure 1.**
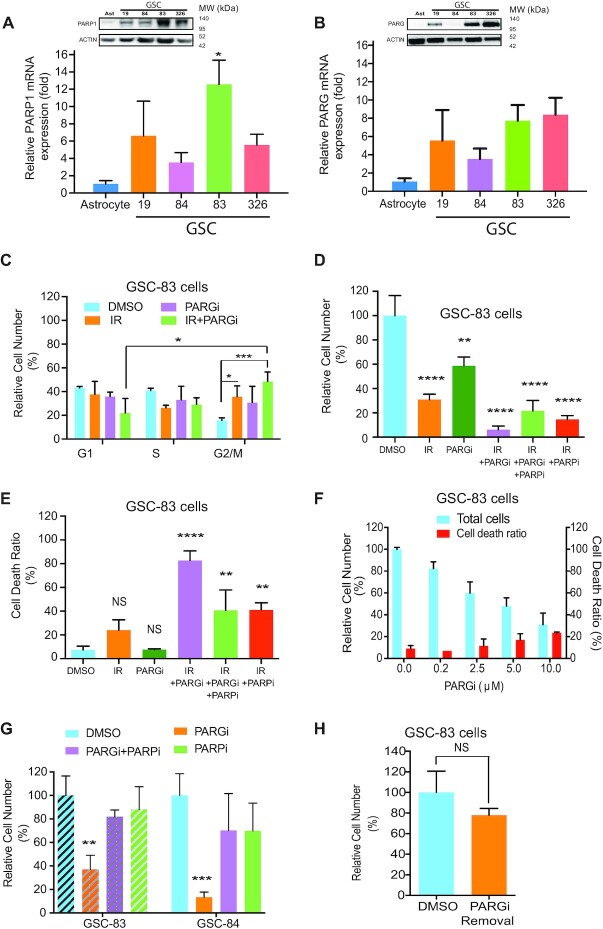
Upregulated PARP1 expression in glioma stem cells—impact on PARG inhibitor response. (**A**) PARP1 mRNA levels from representative glioma stem cells were analyzed by qRT-PCR and normalized to that of normal human astrocytes, (*P < 0.05); insert: Immunoblot analysis of PARP1 among representative GSC cells and normal human astrocyte cells. β-Actin was used as the loading control. (**B**) PARG mRNA levels from representative glioma stem cells were analyzed by qRT-PCR and normalized to that of normal human astrocytes; insert: immunoblot analysis of PARG among representative GSC cells and normal human astrocyte cells. β-Actin was used as the loading control. (**C**) Percentage of GSC-83 cells in G1, S or G2/M phase after treatment with vehicle (DMSO), PARGi (2.5 μM), IR (3 Gy) or IR+PARGi (*P < 0.05, **P < 0.01). (**D**) Relative number of GSC-83 cells (%), normalized to vehicle control, 5-day after treatment: vehicle (DMSO), IR (5 Gy), PARGi (10 μM), IR+PARGi, IR+PARGi+PARPi or IR+PARPi (ABT-888, 10 μM) (**P < 0.01, ****P < 0.0001 related to DMSO). (**E**) Percentage of non-viable GSC-83 cells on the 5th day after treatment: vehicle (DMSO), IR (5 Gy), PARGi (10 μM), IR+PARGi, IR+PARGi+PARPi or IR+PARPi (ABT-888, 10 μM) (NS = not significant, **P < 0.01, ****P < 0.0001). (**F**) Relative number of GSC-83 cell following 5-day PARGi treatment at the doses indicated (left) and percentage of non-viable GSC-83 cells (%) after 5-day PARGi treatment (right), at the doses indicated. (**G**) Relative cell number of GSC-83 cells (left) or GSC-84 cells (right) after a 5-day treatment of DMSO, PARGi (10 μM), PARGi plus PARPi or PARPi (10 μM), normalized to DMSO as 100% (**P < 0.01, ***P < 0.001). (**H**) After 5-day treatment of GSC-83 cells with DMSO or PARGi (10 μM), cells were washed twice with PBS and then the same number of live cells from each treatment were seeded and cultured in growth medium without any treatment for an additional 5 days. The relative number of cells following the initial PARGi treatment is normalized to the cells seeded from those following the initial DMSO treatment (NS = not significant).

While there are many significant gene expression differences among the two major subtypes of glioma stem cells (GSCs) (Proneural, PN; Mesenchymal, MES), as we have described (11), the expression of PARP1 is seen as a major genotypic difference when comparing astrocytes to both types of GSCs (PN and MES). The expression of PARP1 mRNA is highly elevated in all GSC cell lines we have tested, as compared to normal astrocytes (Supplementary Figure S1A). Upregulation of PARP1 is observed at both the mRNA and protein level in both MES (GSC-83, GSC-326) and PN (GSC-19, GSC-84) GSCs (Figure 1A). This is consistent with the upregulation of many DNA damage response genes in GSCs, including PARP1 (12), that corresponds to poorer overall survival in both high-grade GBM (Supplementary Figure S1C) and grade III GBM (Supplementary Figure S1D). While PARP1 is responsible for DNA damage-induced poly-(ADP-ribose) (PAR) synthesis, PARG is the primary enzyme responsible for degrading the PAR polymer. We therefore also evaluated PARG expression in GSC cells, as compared to astrocytes. Similar to PARP1, both PARG mRNA and protein is upregulated in most of the GSCs evaluated (Figure 1B and Supplementary Figure S1B).

Given the elevated level of oxidative damage, replication stress and concomitant PARP1 activation in GSCs (43), we rationalized that the increase in PARP1 expression would lead to enhanced DNA damage and replication-stress induced PARP1 activation. The resulting increase in PAR and PAR-induced cell death (13) could reveal a survival dependence on the activity of the major PAR-degrading enzyme, PARG. A standard therapeutic approach for glioma is ionizing radiation (IR) and so we first evaluated the impact of the PARG inhibitor PDD00017273 on the IR response of GSCs. IR treatment of GSCs yields a dose-dependent increase in genomic DNA damage, as shown using the CometChip assay (42) (Supplementary Figure S1E). Whereas we observed low levels of PAR at the highest IR dose (5Gy) (Supplementary Figure S1F, lane 3 and S1Q), PARGi pre-treatment (2.5 μM, 30 min) of GSC-83 cells promoted robust accumulation of PAR post-IR treatment (Supplementary Figure S1F, lanes 4–8). When evaluated over a time-course, we find that GSCs (GSC-83 cells) treated with IR and continuous exposure to a non-toxic dose of PARGi (2.5 μM) shows PAR accumulation as early as 5 min after treatment with a peak around 15–30 min, and then gradually reduced to basal levels after 120 min (Supplementary Figure S1G, right panel). As above, minimal PAR was observed in the absence of PARGi (Supplementary Figure S1G, left panel), likely due to the elevated expression of PARG in these GSCs. The increase in PAR following IR+PARGi co-treatment correlated with an increase in genomic DNA damage in both GSC-83 cells (Supplementary Figure S1I) and GSC-326 cells (Supplementary Figure S1J), implicating PARGi treatment hinders the repair of IR-induced DNA damage.

PARGi treatment caused a minimal impact on cell cycle distribution, while the IR+PARGi co-treatment significantly increased G2/M arrest (Figure 1C and Supplementary Figure S1H), compared to IR alone or PARGi alone. In-line with the observed G2/M arrest, the IR+PARGi co-treatment resulted in the strongest effect on cell growth, as compared to IR or PARGi alone. Further, the cytotoxic and DNA damage effect of the IR+PARGi combination was strongly suppressed by pre-treatment with the PARP1/PARP2 inhibitor ABT-888 (PARPi; Veliparib, 10 μM) in several GSCs as well as the model glioma cell line, LN428 (Figure 1D,E, Supplementary Figure S1K–M). Note that we observe no significant change in cellular ATP levels, as compared to the DMSO control under these treatment conditions (Supplementary Figure S1N). Overall, these initial studies suggest that effective PARGi-induced cancer cell targeting requires a cell treatment or cellular genetic background that predisposes to enhanced PARP1/PARP2 activation.

Given the elevated levels of replication stress in cancer, particularly glioma (44,45), PARP1 activation at the replication fork should be robust. Therefore, we evaluated the impact of direct PARG inhibition (PARGi) on GSC proliferation and viability. PARGi treatment suppressed GSC MES subtype growth (GSC-83) by as much as 70% but induced only a small percentage of cell death at the highest dose (10 μM) (Figure 1F). Inhibition of PARP1/2 by pre-treatment with ABT-888 (Veliparib, 10 μM) blocked PAR accumulation due to PARGi treatment (Supplementary Figure S1, Panel O). ABT-888, with the lowest PARP trapping capacity among the clinical PARP inhibitors (46), was used to avoid additional toxicity from PARP trapping. When combined with PARGi treatment, ABT-888 fully rescued the suppression of GSC proliferation induced by PARGi in multiple GSCs (Figure 1G), confirming that the GSC/PARGi phenotype depends on the activity of PARP1/2. Similar results were seen for the PN subtype GSC line (GSC-84), also with low accumulation of PAR and a minimal increase in γ-H2AX expression (Supplementary Figure S1, panel P). When all four GSC cell lines were treated with PARGi for 1 h, the different levels of PAR observed may suggest additional mechanisms for the regulation of PARP1 activity and the overall accumulation of PAR (Supplementary Figure S1R) (47).

However, the PARGi impact on GSC proliferation induced only a small percentage of cell death at the highest dose (10 μM) (Figure 1F). Furthermore, when the PARGi was removed (after 5 days of continuous PARGi treatment) followed by re-culturing the washed cells for an additional 5 days, there was no significant difference in the proliferative status of the cells when comparing the washed cells pre-treated with DMSO or PARGi (Figure 1H). These results suggest that although PARG inhibition suppresses GSC proliferation, the effect was mostly cytostatic and therefore unlikely to be an effective approach to affect GSC or glioma tumor survival unless PARP1 activation is enhanced.

### Increased cellular NAD^+^ promotes spontaneous PARP1 activation and replication fork suppression

The lack of a cell-killing effect by PARG inhibitor treatment of GSCs, regardless of the elevated levels of PARP1 protein, is cofounding. Since PARGi response is most robust when in concert with PARP1 activation and PAR formation, it is therefore not surprising that PARGi/IR co-treatment promotes cancer cell death (Figure 1). Given that the PARP1 substrate, NAD^+^, is considered a base excision repair (BER) and single-strand break repair (SSBR) regulatory factor and that PARP1 activation can be modulated by changes in cellular levels of NAD^+^ (14,15), we reasoned that PARP1 activation potential, at the replication fork, may be regulated, in part, by the available level of cellular NAD^+^. If NAD^+^ levels are insufficient to promote PARP1 activation in response to replication stress, this may explain why PARG inhibitors alone have only a cytostatic effect on GSCs (Figure 1).

**Figure 2.**
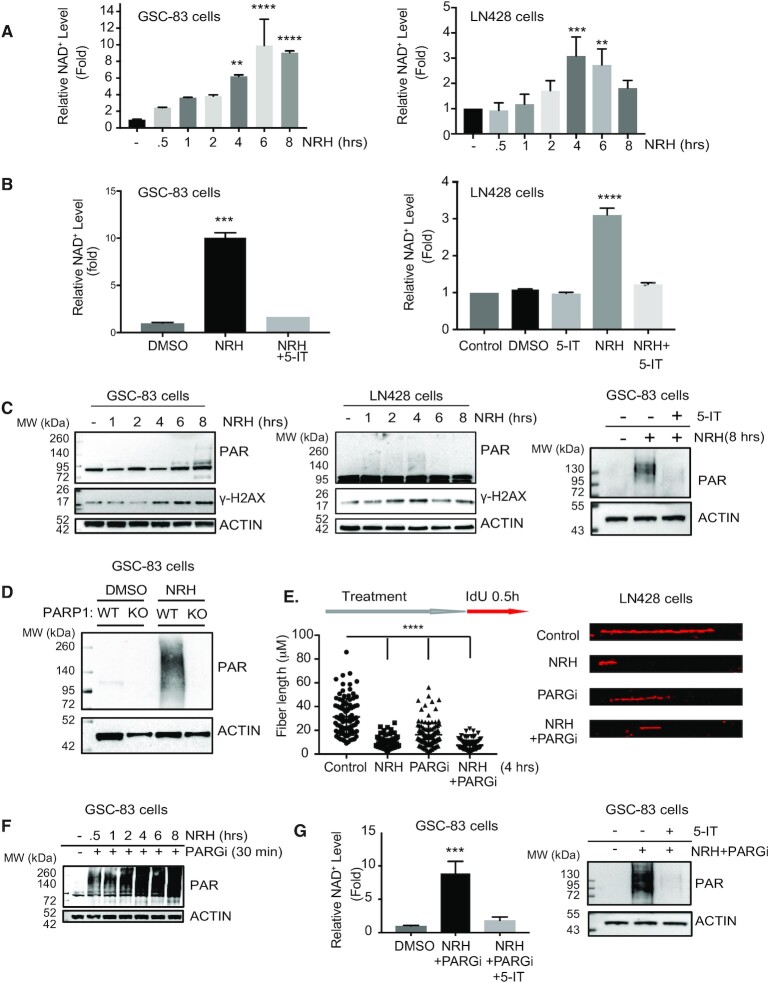
Dihydronicotinamide riboside (NRH) increases cellular NAD^+^ levels and promotes spontaneous PAR formation to suppress replication in GSCs and glioma cells. (**A**) Total cellular NAD^+^ levels in GSC-83 cells (left panel) or LN428 glioma cells (right panel) after treatment with NRH (100 μM) for the time periods indicated, normalized to the NAD^+^ level of cells treated with DMSO; **P < 0.01, ***P < 0.001,****P < 0.0001, related to control. (**B**) Total cellular NAD^+^ levels in GSC-83 cells (left panel) or LN428 glioma cells (right panel) after treatment with NRH (100 μM), the adenosine kinase inhibitor, 5-Iodotubercidin (5-IT) or NRH+5-IT for 4 h, normalized to the NAD^+^ level in cells treated with DMSO; ***P < 0.001,****P < 0.0001, related to control. (**C**) PAR (poly-ADP-ribose) and γ-H2AX immunoblot analysis of total cell lysates (GSC-83 cells, left panel; LN428 cells, middle panel) after treatment with NRH (100 μM) for the times indicated, as compared to cells treated with DMSO. PAR immunoblot analysis of total cell lysates (GSC-83 cells) after treatment with DMSO, NRH (100 μM) or NRH + 5-IT (right panel). For all, β-Actin was used as the loading control. (**D**) PAR (poly-ADP-ribose) immunoblot analysis of total cell lysates (GSC-83/WT or GSC-83/PARP1-KO cells, as indicated) after treatment with NRH (100 μM) for 4 h, as compared to cells treated with DMSO. β-Actin was used as the loading control. (**E**) DNA fiber analysis as a measure of replication (indicated as DNA fiber length) in LN428 cells following treatment with NRH (100 μM), PARGi (10 μM) or NRH plus PARGi for 4 h as compared to untreated cells (control). After 4 h of treatment with NRH, IdU (25 μM) was added to the media for 30 min. Following cell lysis, naked DNA was combed on coverslips followed by immunostaining of DNA fibers that were labeled with IdU. Coverslips were imaged by confocal microscopy and DNA fiber length was measured and plotted (left panel). A representative DNA fiber image of each treatment is shown (right panel) (****P < 0.0001). (**F**) PAR immunoblot analysis from total cell lysates (GSC-83 cells) after treatment with NRH (100 μM) + PARGi (10 μM) for the times indicated. β-Actin was used as the loading control. (**G**) Total cellular NAD^+^ levels in GSC-83 cells (left panel) after treatment with NRH (100 μM) +PARGi (10 μM) or NRH+PARGi+5-IT, normalized to the NAD^+^ level of cells treated with DMSO; ***P < 0.001. PAR immunoblot analysis (right panel) from total cell lysates (GSC-83 cells) after treatment with NRH+PARGi or NRH+PARGi+5-IT (right panel). β-Actin was used as the loading control.

Stressed cells can be treated with NAD-precursors to restore suppressed cellular NAD^+^ levels and promote PARP1-mediated BER/SSBR (15,16). Here, we evaluated a series of NAD-precursor molecules (nicotinomide riboside, NR; nicotinic acid riboside, NAR; dihydro-nicotinamide riboside, NRH; dihydronicotinic acid riboside, NARH) to determine the capacity to increase NAD^+^ over basal levels, in the absence of added stress. Only NRH (Supplementary Figure S2A), a reduced form of NR, was able to significantly increase total cellular levels of NAD^+^ in non-stressed cells (Supplementary Figure S2B). The total cellular NAD^+^ levels in both GSCs and the glioma cell line LN428 could be acutely increased by exposure to NRH (100 μM) (48,49), reaching peak levels (6- to 10-fold increase for GSCs, 3- to 4-fold for LN428) from 4 to 8 h following addition (Figure 2A and Supplementary Figure S2C). The basal level of NAD^+^ for all 4 GSC cells were similar yet the mesenchymal GSC subtypes (GSC-83 and GSC-326) showed a much greater increase in NAD^+^ levels after NRH treatment as compared to the proneural GSC subtypes (GSC-19 and GSC-84). Although only speculation, this may be the result of the distinct transcriptional differences between these GSC subtypes (11) (Supplementary Figure S2C). While NR requires the activity of NRK1 for conversion to NAD^+^ (50), the mechanism of NRH conversion to NAD^+^ is unique, promoting rapid increases in NAD^+^ dependent on the activity of adenosine kinase (AK) (49). In-line with this suggested mechanism, inhibition of AK blocks the NRH-induced increase in total cellular NAD^+^ levels (Figure 2B).

We next determined if the NRH-induced increase in NAD^+^ may have an impact on replication stress induced PARP1 activation. Coincident with the NRH-induced peak of NAD^+^ levels, spontaneous increases in low levels of PAR and γH2AX were observed in both the GSCs and LN428 cells following treatment with NRH, and PAR formation was suppressed when AK was inhibited (Figure 2C). The increase in PAR levels following the NRH-induced acute increase in NAD^+^ is primarily dependent on PARP1 (Figure 2D and Supplementary Figure S2D). Consistent with the role of PARP1-mediated PARylation suppressing replication fork progression, the dose and time of NRH treatment that promotes the highest level of NAD^+^ and PAR synthesis also suppresses replication fork progression, similar to the suppressive effect from PARGi. In-line with our model, the greatest effect on replication is seen by the combined NRH+PARGi treatment (Figure 2E). In the presence of NRH and likely due to replication associated PARP1 activation, PARGi treatment promotes a massive increase in time dependent PAR accumulation in GSCs (Figure 2F and Supplementary Figure S2I). However, the increase in PAR is not seen when cells are pre-treated with NR (Supplementary Figure S2E) nor when PARP1 is deficient or inhibited (Supplementary Figure S2F). While NRH promotes as much as a 10-fold increase in NAD^+^ levels in GSCs, PARG inhibition does not impact the total NRH-induced increase in the NAD^+^ pool (Supplementary Figure S2G) and the elevated NAD^+^ levels or PAR levels after NRH+PARGi in GSCs or LN428 cells is still dependent on AK activity (Figure 2G and Supplementary Figure S2H). Given the observed increase in PAR upon co-treatment with NRH and PARGi and the NRH-induced impact on replication, we next evaluated whether PARG inhibitor effects on cell viability greatly depend on cellular NAD^+^ levels and the resulting increased capacity for replication-associated PARP1 activation.

### Selective PARGi-induced cell death in GSCs dependent on NRH-enhanced cellular NAD^+^

**Figure 3.**
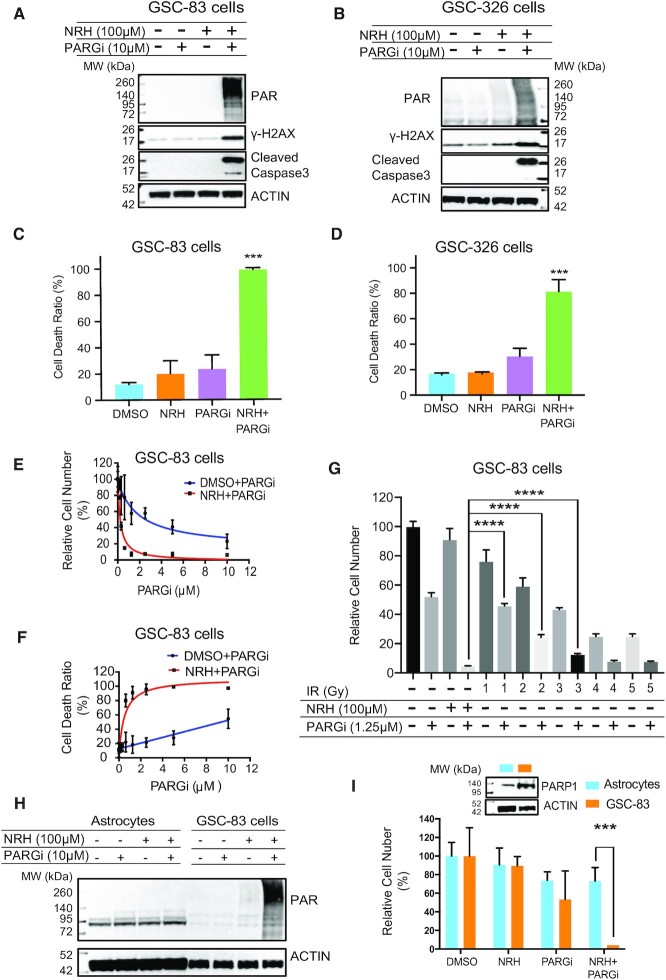
Selective PARGi-induced cell death in GSCs is dependent on NRH-enhanced levels of cellular NAD^+^. (**A**) Immunoblot analysis of total cell lysates for PAR, γ-H2AX and cleaved Caspase-3 after 24 h treatment of GSC-83 cells treated with DMSO, NRH (100 μM), PARGi (10 μM) or NRH+PARGi. β-Actin was used as the loading control. (**B**) Immunoblot analysis of total cell lysates for PAR, γ-H2AX and cleaved Caspase-3 after 24 h treatment of GSC-326 cells treated with DMSO, NRH (100 μM), PARGi (10 μM) or NRH+PARGi. β-Actin was used as the loading control. (**C**) The percentage of dead cells (GSC-83 cells) after 5-day treatment with DMSO, NRH (100 μM), PARGi (10 μM) or NRH+PARGi; (***P < 0.001). (**D**) The percentage of dead cells (GSC-326 cells) after 5-day treatment with DMSO, NRH (100 μM), PARGi (10 μM) or NRH+PARGi; (***P < 0.001). (**E**) The relative number of GSC-83 cells after a 5-day treatment with PARGi or NRH (100 μM) plus PARGi (PARGi doses as indicated), normalized to DMSO. (**F**) The relative number of non-viable GSC-83 cells after a 5-day treatment with PARGi or NRH (100 μM) plus PARGi (PARGi doses as indicated), normalized to DMSO. (**G**) The relative number of GSC-83 cells after a 5-day treatment with NRH (100 μM), PARGi (1.25 μM), NRH plus PARGi, IR at the indicated dose (1–5 Gy) or PARGi (1.25 μM) plus IR at the indicated dose (1–5 Gy), normalized to the control; ****P < 0.0001. (**H**) Immunoblot analysis of PAR in lysates of astrocytes or GSC-83 cells after 2 h treatment with DMSO, NRH (100 μM), PARGi (10 μM) or NRH+PARGi. β-Actin was used as the loading control. (**I**) The relative number of astrocytes or GSC-83 cells after a 5-day treatment with DMSO, NRH (100 μM), PARGi (10 μM) or NRH+PARGi (***P < 0.001). Insert: Immunoblot analysis of PARP1, comparing astrocytes and GSC-83 cells. β-Actin was used as the loading control.

We observed a clear synergistic increase in PAR accumulation in response to dual NRH/PARGi treatment, with a coincident increase in the expression of γ-H2AX and of cleaved caspase 3, 24 h post exposure in both GSC-83 and GSC-326 cells (Figure 3A,B). In addition to the increase in cleaved caspase 3, we observed a >30-fold increase in caspase 3/7 activity (Supplementary Figure S3A), suggestive of a strong apoptotic response that may translate to increased cell killing. Consistently, we find that while PARG inhibition (10 μM) alone promotes <30% cell death in both GSC-83 and GSC-326 cells, the addition of a non-toxic dose of NRH (100 μM), and the resulting increase in cellular NAD^+^ levels, results in near 100% cell killing in multiple glioma stem cell lines (Figure 3C, D). A PARGi dose–response analysis showed that NRH plus low doses of PARGi (<1 μM) for 5 days results in a decrease in total cell number (Figure 3E) and a 30- to 40-fold decrease in cell viability (Figure 3F). Both the elevated level of PAR and the increase in cell death, when combining NRH and PARG inhibition, were blocked by ABT-888 mediated PARP1/2 inhibition (Supplementary Figure S3B and C). We also compared the efficacy of the suppression of GSC-83 cell growth by treatment of IR+PARGi to NRH+PARGi. Using the near IC_50_ dose for the PARGi (1.25 μM) and a non-toxic does of NRH (100 μM), the NRH+PARGi treatment induced a significantly stronger suppression of GSC-83 cell growth than IR+PARGi (Figure 3G).

Enhanced GSC and tumor cell death is of little value if normal cells are equally sensitized. To that end, we compared GSCs to normal astrocytes, probing the levels of PAR, by immunoblot, induced by the NRH/PARGi co-treatment. Importantly, we found no increase in PAR nor loss of viability in the actively dividing astrocyte cultures, likely due to the extremely low levels of PARP1 expression (Figure 3H,I). Together, we find that treatment of NRH, to acutely raise cellular NAD^+^ levels, supports robust PARP1 activation in GSC/tumor cells in response to replication stress. In combination with a potent PARG inhibitor, the enhanced PARP1 activation triggers selective tumor cell apoptosis, with little effect on normal cells such as astrocytes.

### PARGi-induced S-phase arrest and checkpoint activation require enhanced cellular NAD^+^ from NRH exposure

**Figure 4.**
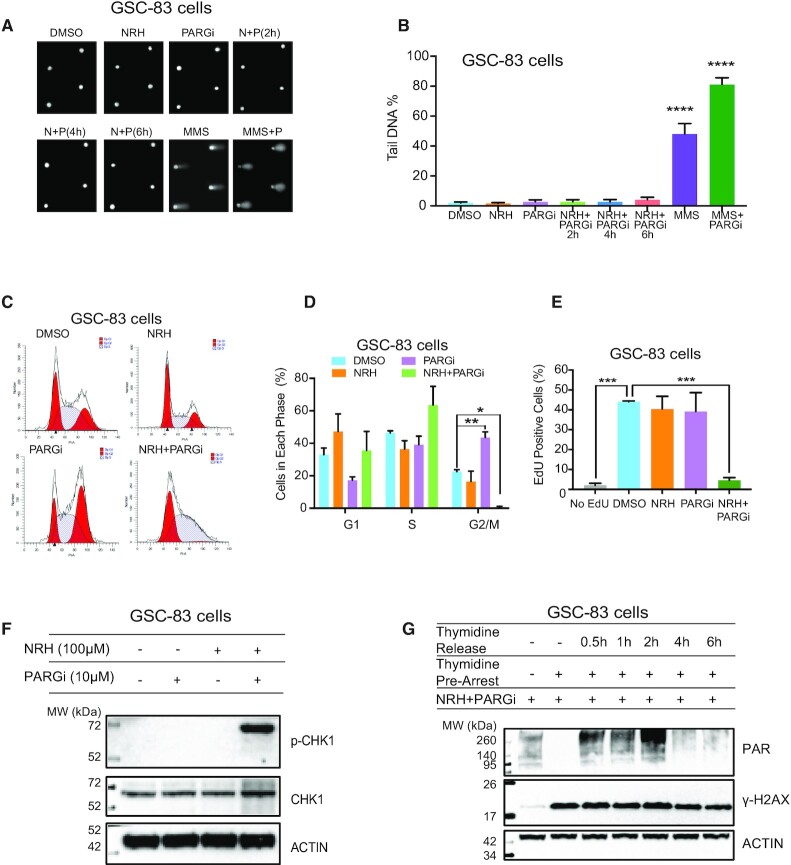
PARGi-induced S-phase arrest and checkpoint activation requires enhanced cellular NAD^+^ from NRH exposure. (**A** and **B**) Representative Comet images (A) and quantification of genomic DNA damage (B) resulting from cellular treatment with DMSO, NRH (100 μM), PARGi (10 μM) or NRH+PARGi (N+P) for the duration indicated. GSC-83 cells were also exposed to MMS (1 mM) or MMS+PARGi (MMS+P) for 30 min. DNA damage was evaluated by the CometChip assay, reported as % Tail DNA; ****P < 0.0001 as compared to the control, DMSO. (**C**) Flow cytometry cell cycle analysis of GSC-83 cells 24 h after treatment with DMSO, NRH (100 μM), PARGi (10 μM) or NRH+PARGi. (**D**) Quantification of the cell cycle analysis of GSC-83 cells 24 h after treatment with DMSO, NRH (100 μM), PARGi (10 μM) or NRH+PARGi (*P < 0.05, **P < 0.01), showing the percentage of cells in each cell cycle phase (three separate experiments). (**E**) Percentage of EdU positive cells (GSC-83 cells) 24 h after treatment with DMSO, NRH (100 μM), PARGi (10 μM) or NRH+PARGi followed by 2 h EdU incorporation (***P < 0.001). (**F**) Immunoblot analysis of CHK1 and phosphorylated CHK1 (p-CHK1) 24 h after treatment with DMSO, NRH (100 μM), PARGi (10 μM) or NRH+PARGi. β-Actin was used as the loading control. (**G**) Immunoblot analysis of PAR and γ-H2AX in whole cell lysates prepared from GSC-83 cells treated with NRH (100 μM) + PARGi (10 μM) for 1 h under normal growth conditions, arrested by thymidine or released from thymidine arrest for 0.5, 1, 2, 4 or 6 h. β-Actin was used as the loading control.

The selective and enhanced PAR-induced cellular lethality following co-treatment of a PARG inhibitor with NRH can feasibly result from either enhanced global DNA damage and/or enhanced replication stress in cancer cells, both known to activate PARP1. This is evident from the observed strong increase in the expression of γ-H2AX after co-treatment (Figure 3A,B), considered a marker of both DNA damage (51) and replication stress (52). To differentiate these two possibilities, we evaluated global genomic levels of DNA damage by the CometChip assay (42). It is noted here that the NRH/PARGi combination does not induce measurable DNA damage, as compared to methyl methanesulfonate (MMS) + PARGi treatment (Figure 4A,B; Supplementary Figure S4A, S4B). However, the same treatment results in strong activation of the apoptosis pathway (Figure 3A,B; Supplementary Figure S3A), suggesting that PARP1 activation, under these conditions, is likely the result of replication stress that would impact cell cycle distribution. Consistent with this mechanism, the NRH/PARGi co-treatment fully arrested GSC-83 cells in the S-phase (Figure 4C,D); clearly a very different response as compared to PARG inhibitor treatment following irradiation (Figure 1C). We find that with the NRH/PARGi treatment, there is an accumulation of cells in S-phase and no measurable cells in G2/M, suggestive of an intra-S-phase checkpoint (Figure 4C,D).

The block to replication, minimally seen with NRH alone, is further enhanced when combined with a PARG inhibitor. While as much as 40% of the glioma stem cells showed positive EdU incorporation following either DMSO, NRH or PARG inhibitor treatment, there was only a minimum EdU signal (< 5%), close to the negative control (2%), in the NRH/PARGi-treated cells (Figure 4E), indicating a block to DNA replication. The induction of an intra S-phase arrest was further demonstrated by the activation of the S-phase checkpoint protein CHK1 (28), with a strong increase in phosphorylated CHK1 levels 24 h after NRH/PARGi treatment (Figure 4F). Consistent with the rapid accumulation of PAR synthesis and the onset of the S-phase checkpoint following NRH/PARGi treatment, we observed robust nuclear accumulation of PAR, evaluated by a fluorescent probe for PAR, a highly sensitive live-cell real-time probe for the analysis of poly-ADP-ribosylation (LivePAR) (14). This genetically encoded WWE-domain/EGFP probe forms nuclear foci within 4 h of NRH/PARGi treatment (Supplementary Figure S4C). Both the LivePAR probe and PAR (as evaluated by immunofluorescent staining) predominantly co-localizes with PCNA (Supplementary Figure S4D and S4E), suggesting that the PAR foci are likely at sites of stalled replication. The increase in PAR in response to the NRH/PARGi treatment is consistent with a response to replication stress. This would suggest that replication is required for the accumulation of PAR under these treatment conditions. To test this, we blocked DNA replication in the GSC-83 cells (72 h, 2 mM thymidine treatment) (Supplementary Figure S4F). Such a block to replication shows no observable toxicity and removal of thymidine by PBS washing releases 80–90% of the arrested cells into S-phase within a few hours (53). The arrested GSC-83 cells or the cells released from the block (for 0.5, 1, 2, 4 and 6 h) were then co-treated with NRH and PARGi for 1 h, followed by PAR analysis. While a strong PAR signal was detected within 30 min after the release from the thymidine block (Figure 4G), no PAR signal was detected for the arrested cells (no replication). Together, these results indicate co-treatment of NRH and PARGi responds to replication stress at the replication fork, resulting in a block to DNA replication and activation of an S-phase checkpoint.

### Replication associated PARP1 activation coordinates BER/SSBR pathway engagement and PAR-induced assembly of the replication initiation complex

**Figure 5.**
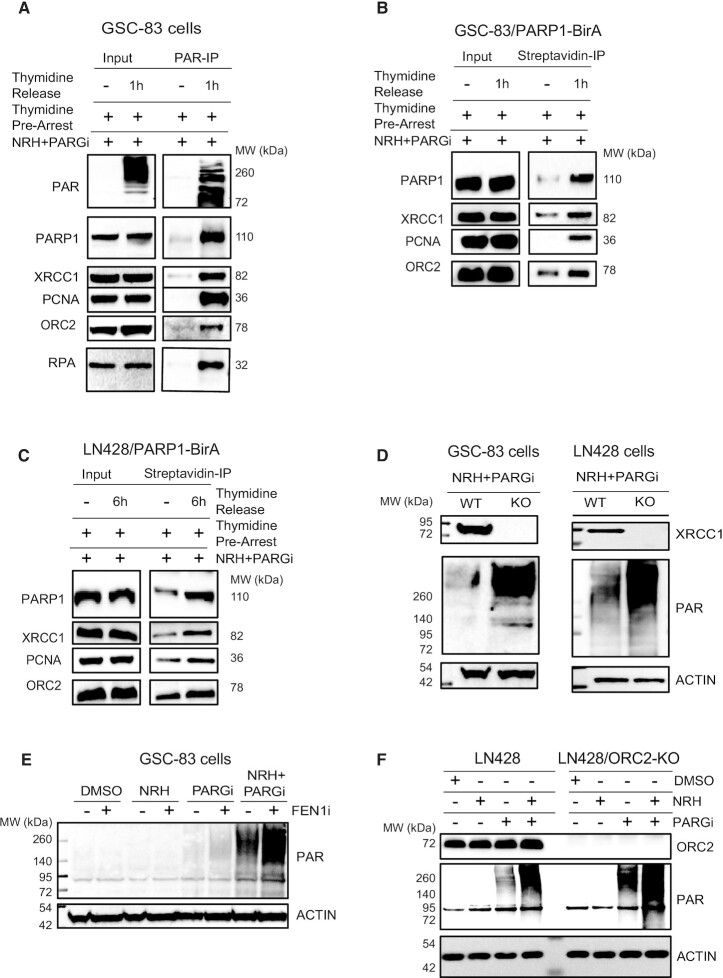
Replication-associated PARP1 activation coordinates BER/SSBR pathway engagement and PAR-induced assembly of replication complex proteins. (**A**) Immunoblot analysis of PAR, PARP1, XRCC1, PCNA, ORC2 and RPA from whole cell lysates (input) and after captured by PAR-IP using an anti-PAR resin, isolated from arrested or released GSC-83 cells, co-treated with NRH (100 μM) + PARGi (10 μM) for 1 h. (**B**) Immunoblot analysis of PARP1, XRCC1, PCNA and ORC2 of whole cell lysates (input) and of biotinylated proteins captured by streptavidin-IP after GSC-83/PARP1-BirA cells were arrested or released and then treated with NRH (100 μM) + PARGi (10 μM) for 1 h. (**C**) Immunoblot analysis of PARP1, XRCC1, PCNA and ORC2 of whole cell lysates (input) and of biotinylated proteins captured by streptavidin-IP after LN428/PARP1-BirA cells were arrested or released and then treated with NRH (100 μM) + PARGi (10 μM) for 6 h. (**D**) Immunoblot analysis of XRCC1 and of PAR from lysates of GSC-83/WT or GSC-83/XRCC1-KO cells treated with NRH (100 μM) + PARGi (10 μM) for 1 h (left panel) and LN428/WT or LN428/XRCC1-KO treated with NRH (100 μM) + PARGi (10 μM) for 6 h (right panel). β-Actin was used as the loading control. (**E**) Immunoblot analysis of PAR after GSC-83 cells were pretreated with FEN1 inhibitor for 1 h then treated with DMSO, NRH (100 μM), PARGi (10 μM) or NRH+PARGi for an additional 1 h. (**F**) Immunoblot analysis of OCR2 and PAR after LN428 or LN428/OCR2-KO cells were treated with DMSO, NRH (100 μM), PARGi (10 μM) or NRH+PARGi for 4 hours.

The accumulation of PAR in GSC and glioma tumor cells that results from co-treatment with NRH and a PARG inhibitor is coupled with DNA replication, consistent with PARP1 activation, in response to replication stress, regulating replication fork progression (29). Once activated, the resulting poly-ADP-ribose (PAR) facilitates protein complex recruitment to facilitate repair and activate signal transduction pathways, as we have shown for the regulation of BER protein complex formation and the induction of a block to glycolysis (15–17). Expecting novel proteins to be involved in and recruited by PAR, produced by replication associated PARP1 activation, we co-treated the thymidine arrested cells (DNA replication arrested) or released cells (DNA replicating) with NRH/PARGi. The PAR-modified and PAR-bound proteins were then captured using a specific PAR binding resin, comprised the Af1521-macrodomain bound to agarose, as depicted in Supplementary Figure S5C. This approach is preferred so as to reduce the possibility of false positive results from direct co-immunoprecipitation of the PARP1 protein. The Af1521-macrodomain bound proteins were then probed by immunoblot, focusing on those involved in BER/SSBR (54) and those predicted to be involved in the PARP1-associated replication complex. Two important proteins in the BER/SSBR pathway, PARP1 and XRCC1, as well as the DNA replication related proteins PCNA, ORC2 and RPA, were captured after the co-treatment and only from the replicating cells (Figure 5A). To rule out the possibility of inhibition of PARP1 activity by thymidine, however unlikely, we treated the thymidine arrested GSC-83 cells with the DNA alkylating agent MMS, a strong inducer of DNA damage induced PARP-activation (55). As expected, addition of the PARGi resulted in a strong PAR signal in the non-replicating, thymidine-treated cells due to MMS treatment (Supplementary Figure S5B). This re-enforces the concept that the response to replication stress activates PARP1 to initiate replication associated BER/SSBR and replication arrest.

As a complement to this approach, to determine whether the PARP1/PAR complex associated proteins are not limited to a protein–protein interaction in cell lysates, we developed a PARP1/BioID system (Supplementary Figure S5D) to validate PARP1-interacting proteins (56–58). Expressing a PARP1 biotin ligase (BirA-R118G) fusion (PARP1-BirA) allows for the temporal analysis of PARP1 protein complexes modulated in response to PARP1 activation, in this case at the replication fork. In addition to finding uniform biotinylation of PARP1, the level of the BER/SSBR protein XRCC1 and the DNA replication related proteins PCNA and ORC2 were increased after PARP1 activation and PAR accumulation from co-treatment of NRH and PARGi in both the GSC-83 and LN428 cells (Figure 5B,C). It is expected, therefore, that these replication-associated BER/SSBR protein complexes are essential for PARP1-mediated repair. In support of this, PAR accumulation is significantly stronger in XRCC1-KO cells (co-treated with NRH and PARGi) as compared to the wild-type (WT) cells in both cell lines (Figure 5D).

**Figure 6.**
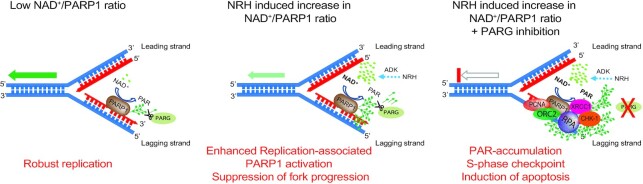
Model of replication-associated PARP1 activation, defining PARGi-induced replication arrest and dependence on NRH-enhanced levels of cellular NAD^+^. The models depict a hypothetical binding site for PARP1 on the lagging strand, post-replication, likely associated with Okazaki-fragment processing (although the PARP1-binding site is not defined): Left panel: A low NAD^+^/PARP1 ratio can suppress PARP1 activation potential, allowing robust DNA replication with minimal PAR accumulation; Middle panel: Alteration of the NAD^+^/PARP1 ratio promotes elevated replication-associated PARP1 activation and a suppression of replication fork progression; Right panel: Combination of (i) NRH to enhance cellular NAD^+^ levels and (ii) PARG inhibition, leads to rapid accumulation of PAR resulting from replication-associated PARP1 activation. PAR accumulation at the replication fork leads to complexation of PARP1 with BER/SSBR proteins such as XRCC1 and replication proteins including RPA, PCNA and ORC2, triggering the onset of CHK1 phosphorylation, an intra S-phase checkpoint and the onset of apoptosis.

To further confirm involvement of DNA replication related BER, we arrested DNA replication in GSC-83/SCR and GSC-83/XRCC1-KO cells (thymidine-block) and then co-treated the cells with NRH/PARGi. There is no detectable PAR accumulation in the WT cells after DNA replication arrest, consistent with the requirement for DNA replication to promote PAR accumulation after the NRH/PARGi treatment (Figure 5A). However, there was a weak but detectable level of PAR accumulation in the XRCC1-KO cells, suggesting possible low levels of global BER/SSBR activity in addition to replication-associated BER/SSBR (Supplementary Figure S5A). Because increased PAR at sites of DNA replication may also relate to the repair of un-ligated Okazaki fragments (23), we inhibited FEN1, an enzyme involved in long-patch BER (59) and in processing of the 5’ end of Okazaki fragments (60). As predicted, we found increased levels of PAR following FEN1i/NRH/PARGi treatment, as compared to FEN1 inhibition alone (Figure 5E), suggesting that the increased PARP1 activity following NRH-induced increase in cellular NAD^+^ levels is, in part, related to the processing of unligated Okazaki fragments. Further, we find that ORC2, an essential component of the ORC replication-initiation complex, forms part of the multi-protein complex formed in response to PARP1 activation (Figure 5A–C) and the resulting replication arrest. Like XRCC1, loss of ORC2 enhances the level of replication associated PARylation (Figure 5F). While many human cells can tolerate and replicate in the absence of ORC2 (61), the identification of ORC2 as part of the PARP1/PAR complex in response to replication-associated PARylation and activation of BER/SSBR suggests an additional role for ORC2 in replication stress. Together, we find that accumulation of PAR due to replication associated PARP1 activation engages both key BER/SSBR proteins and replication related proteins (Figure 6), likely to facilitate repair in response to replication stress.

## DISCUSSION

A hallmark of malignant high-grade gliomas (HGGs), such as glioblastoma multiforme (GBM), is their intrinsic resistance to current therapies that leads to extremely poor clinical outcomes (62). HGG tumors are composed of heterogeneous tumor cell populations including tumor cells with stem cell properties termed glioma initiating/propagating cells or glioma stem cells (GSCs) (63) that contribute to therapeutic resistance (64,65). A noted molecular feature of GSCs is elevated replication stress (44,45,66).

Both endogenous and exogenous sources of genotoxic stress pose a significant challenge to the DNA replication machinery (20). Together, these factors lead to the common term replication stress (67). PARP1 responds to replication stress (20), and recently, mechanistic models of replication stress have emerged that suggests several critical roles for PARP1 that likely involves repair of base lesions and DNA breaks. The number of different models proposed to-date in response to the many types of replication stress are numerous (20,67–69). However, PARP1 has been suggested to have several possible roles. Regarding replication fork remodeling and processing, PARP1 interacts with and signals to RECQ1 to suppress replication restart (22). Further, PARP1 is activated, likely associated with recognition of Okazaki fragments, to recruit XRCC1 (23,24) and together with BRCA2, blocks fork degradation by MRE11 (26,27).

Nicotinamide adenine dinucleotide is an essential metabolite and co-enzyme present in cells in both an oxidized form (NAD^+^) and reduced form (NADH) where it is involved in cell metabolism (13). As we have shown, loss of NAD^+^ leads to a defect in mitochondrial functions such as oxidative phosphorylation (17). Beside its role in energy production, the second major role for the NAD^+^ coenzyme is as a substrate of the ADP-ribosyltransferase enzymes (PARPs) (70) and of the NAD-dependent deacetylases (sirtuins) (71). In this regard, alterations in NAD^+^ levels can have a major impact on genome integrity since both PARPs and sirtuins have a significant impact on several DNA repair mechanisms and genome stability (72–80). As we have shown, decreased NAD^+^ can modulate the cellular response to DNA damage, over-coming cellular resistance to select genotoxins (81). Further, suppression of NAD^+^ biosynthesis will block cellular PARP1 activation (17), leading to a defect in cellular DNA repair capacity (15) and suppression of BER/SSBR complex formation (14,15) while increasing NAD^+^ bioavailability will enhance repair protein complex assembly (14).

Here, we found that GSCs express elevated levels of PARP1 and PARG. Since activated PARP1 produces PAR, we reasoned that blocking PARG, the primary enzyme to degrade PAR, would enhance PAR-induced cell death (17) in GSCs. Unlike recent studies that suggested PARGis alone would be effective in selectively killing cancer cells (33,35,82), PARGi treatment of GSCs resulted in minimal but variable levels of cell death unless the PARGi is combined with a DNA damaging agent (e.g., an alkylating agent or radiation), as found in other reports (32,34,83,84). Importantly, this is consistent with our finding that glioma cells and GSCs need to be supplemented with NAD^+^-precursors to raise cellular NAD^+^ levels sufficient for robust PARP-activation in response to DNA damage. We show that treatment with the NAD^+^ precursor NRH (85) increases the NAD^+^ cellular level in GSCs by as much as 10-fold and allows higher PARP1-activation potential in response to DNA damage or dependent on replication. We note that the NRH-induced increase in NAD^+^ is dependent on adenosine kinase activity, as reported (49). This increase in NAD^+^ also promotes replication dependent PARP1 activation and a block to replication fork progression. This is consistent with recent reports that glioma cells are defective in NAD^+^ biosynthesis, especially for those with IDH1 somatic mutations (86) and suggests a major factor in the regulation of PARP1 signaling in GSCs and glioma cells may be related to a defect in NAD^+^ biosynthesis.

Since it was reported that variations in cellular NAD^+^ levels can alter the proteins that interact with PARP1 (87), we used the BioID approach by expressing PARP1-BirA in glioma cells to determine the spontaneous and NAD^+^-regulated PARP1-interactome. Of the proteins that showed differential biotinylation, many were replication associated proteins, including PCNA and ORC2, as well as XRCC1. Further, using PAR-capture, many of these same proteins were identified, including XRCC1, PCNA, ORC2 and RPA, suggesting that each are part of the PAR-modified protein complex formed in response to replication stress. This suggests that in the absence of exogenous DNA damage, PARP1 plays a critical role at the replication fork by recruiting essential regulator proteins. Importantly, we find that PAR accumulation is regulated by select PARP1 and PAR interacting proteins such as XRCC1 and ORC2. Whereas the involvement of XRCC1 highlights the role of the BER/SSBR pathway in responding to replication stress, the enhanced interaction of PARP1 with PCNA, RPA and ORC2 upon PAR accumulation suggests that replication associated PARP1 activation and assembly with pre-replication complex proteins may be an initiating event that leads to replication arrest and apoptosis, a process that is controlled by PAR synthesis and dynamics and regulated by the PAR degrading enzyme PARG. It is interesting to speculate that the formation of these PAR-driven protein complexes may be the result of liquid-liquid phase separation and the formation of biomolecular condensates regulated by PARylation, as suggested recently (88–90). Such processes have been postulated to promote heterochromatin domains (91) and to stimulate the formation of complexes with the BER protein APE1 (92), among other DNA repair proteins (93), including MDC1 and 53BP1 (94,95).

Importantly, we find that activation of PARP1 at the replication fork is significantly enhanced by increasing NAD^+^ cellular levels, resulting in PARGi induced synthetic lethality in GSCs. Together, this highlights that both PARP1 protein levels and NAD^+^ cellular levels or NAD^+^ biosynthesis proteins may be valuable biomarkers for PARGi response. As we find herein, the elevated levels of PARP1 in GSCs provides selective targeting by NRH/PARGi treatment and documents a novel regulatory mechanism of PARP1 activation capacity through substrate regulation.

## DATA AVAILABILITY

The authors declare that all data supporting the findings of this study are available within the article or from the corresponding author upon request. The microarray data (Fig. S1A,B) is available through the National Center for Biotechnology Information Gene Expression Omnibus (NCBI GEO) under accession number GSE67089.

## Supplementary Material

zcab044_Supplemental_File
