# Supplementary material for: NAD+ bioavailability mediates PARG inhibition-induced replication arrest, intra S-phase checkpoint and apoptosis in glioma stem cells
Source: NAR Cancer. 2021 Nov 17;3(4):zcab044. doi: 10.1093/narcan/zcab044 (PMC8600031; doi:10.1093/narcan/zcab044)
Supplement: zcab044_Supplemental_File [file zcab044_supplemental_file.pdf]

Supplementary Information for:

Title

NAD^+ bioavailability mediates PARG inhibition-induced replication arrest, intra S-phase checkpoint and apoptosis in glioma stem cells

Short Title

NAD^+ bioavailability regulates PARGi efficacy

Authors
Jianfeng Li[1,2], Kate M. Saville[1,2], Md Ibrahim[1,2], Xuemei Zeng[3], Steve McClellan[1], Anusha Angajala[1,2], Alison Beiser[1,2], Joel F. Andrews[1], Mai Sun[3], Christopher A. Koczor[1,2], Jennifer Clark[1,2], Faisal Hayat[1,2], Mikhail V. Makarov[1,2], Anna Wilk[1,2], Nathan A. Yates[3,4], Marie E. Migaud[1,2] and Robert W. Sobol[1,2]*

Affiliations
[1]Mitchell Cancer Institute, University of South Alabama, Mobile, AL 36604, USA
[2]Department of Pharmacology, College of Medicine, University of South Alabama, Mobile, AL 36604, USA
[3]Biomedical Mass Spectrometry Center, University of Pittsburgh Schools of the Health Sciences, Pittsburgh, PA 15213, USA
[4]Department of Cell Biology, University of Pittsburgh School of Medicine, Pittsburgh, PA 15261, USA

*Correspondence: Robert W. Sobol, PhD, Mitchell Cancer Institute, University of South Alabama, 1660 Springhill Avenue, Mobile, AL 36604, USA.
E-mail: rwsobol@southalabama.edu

Key Words
Glioma stem cell, PARG inhibitor, PAR, NAD^+, NAD^+-bioavailability, NRH, poly-(ADP-ribose)

Supplemental Materials and Methods

Cells and cell culture conditions

Glioma stem cells (GSCs) derived from high-grade glioma (HGG) samples, both the MES subtype (GSC-83, GSC-326) and the PN subtype (GSC-19, GSC-84), were described by us previously (1). GSCs were cultured in suspension in GSC growth medium [DMEM-F12 (Cat# 10565, Life Technologies) supplemented with B27 (1:50), heparin (5mg/mL), basic FGF (bFGF) (20ng/mL), and EGF (20ng/mL)] in 100x20 mm Petri dishes (Cat# 0875711Z, Fisher Scientific) at 37°C and 5% CO_2. Growth medium was changed every 3 or 4 days as needed. LN428 glioblastoma cells and derived cell lines were cultured in alpha MEM supplemented with 10% heat-inactivated FBS (FBS-HI), L-glutamine, antibiotic/antimytotic and gentamycin, as we have described (2-4) and were cultured at 37°C and 5% CO_2. Norman human Astrocytes were obtained from Lonza (Catalog #: CC-2565) and were cultured in AGM TM Astrocyte Growth Medium BulletKit (Catalog #: CC-3186, Lonza) at 37°C and 5% CO_2. Details for each cell line used herein are in **Supplementary Table S1**.

DNA Microarray Analysis

Comparative analysis of mRNA expression, using the Human U219 Array Strip and the Affymetrix GeneAtlas system, was described by us previously (1). Microarray analysis for each of the cell cultures (in triplicate) was accomplished with 100ng purified total RNA as the initial material and the corresponding amplified and labeled antisense RNA (aRNA) using a GeneChip 3′ IVT Express kit (Affymetrix), as described by the manufacturer. The resulting aRNA was fragmented as described by the manufacturer. The labeled aRNAs were then mixed with hybridization master mix. The hybridization cocktails were then denatured at 95°C for 5min, followed by 45°C for 5min, and then kept at 45°C until applied to the hybridization tray

(GeneAtlas System; 120 μL hybridization cocktail was transferred into a well of a four-well hybridization tray). The array strip was immersed into a hybridization mixture and incubated in the hybridization station at 45°C for 16 h. After hybridization, the strip was washed and stained in the GeneAtlas Fluidics Station using the GeneAtlas Hybridization, Wash, and Stain Kit (#900720; Affymetrix), and the intensity of each hybridized probe was generated using the GeneAtlas Imaging Station. Raw cell files from the Human U219 Array Strip was analyzed using the "affy" package in R Bioconductor. The raw data were normalized and summarized using the robust multichip average method (RMA). Each gene is represented by one or more probe sets. The probe sets expressing <75 units for all samples were filtered out for the genes with other probe sets expressed (>75 units). The selective filtering was performed to avoid getting rid of any gene altogether. For genes represented by multiple probe sets, the probe set with the highest interquartile range (a descriptive statistic used to summarize the extent of the data spread) was selected to represent the gene. As a result of the filtering procedure, all genes are represented by a single probe set for further statistical analysis.

PARP1 or XRCC1 knockout by CRISPR/Cas9

Guide RNAs (gRNAs) targeting PARP1 exon 1 or exon 2 were designed using the CRISPR Design Tool (5), and as described (6). Guide RNAs (gRNAs) targeting XRCC1 were described by us previously (2). Each separate gRNA was cloned into pLentiCRISPRv2 (7). Details for each vector developed or used herein is described in **Supplementary Table S3**. The sequence of each gRNA target sequence and the oligonucleotides used for the vector development is detailed in **Supplementary Table S3**. The experiment to target PARP1 or XRCC1 was performed as described (7,8). Briefly, the GSC or LN428 cell lines were transduced by lentivirus, as we have described previously (2-4), prepared from the corresponding gRNA expression vectors (kindly provided by Wim Vermeulen, Erasmus MC, Netherlands). Cells

were then seeded to select single-cell clones, and knockout was confirmed by immunoblot analysis of whole-cell lysates with a PARP1 antibody (sc-7447, Santa Cruz) or XRCC1 anntibody (A300-065A, Bethyl). See also **Supplementary Table S2.**

CometChip analysis

The CometChip Platform was described in detail in our earlier report (9). The 30-micron CometChips (glass-backed CometChip cassettes with 1mm agarose and micro-patterned micro-wells each at 30-micron width), a well-former for 96-well assembly, the CometChip Electrophoresis System (CES) and the Comet Analysis Software (CAS) are all available from BioTechne (Minneapolis, MN). For these studies, GSC-83 cells ($1x10^6$) were seeded in a 35mm dish with 2ml medium supplemented with DMSO, PARPi, PARGi or PARPi plus PARGi for 30 minutes. As detailed in the figure legends, in some cases the cells were then irradiated using the X-Rad 320 system at the indicated dosage or were treated with MMS (1mM) for 30 min. After the treatment period, a cell suspension (100µl) was then loaded into each well of the 30-micron CometChip and kept at 4˚C for 30 min to allow the cells to set into the microwells. Next, the CometChip was washed twice with PBS and sealed with low melting point agarose (LMPA; Topvision; ThermoFisher Scientific; 7 ml; 0.75% LMPA/PBS). The CometChip was then submerged in lysis solution with detergent (BioTechne) overnight at 4˚C. Electrophoresis of the CometChip was run under alkaline conditions (pH>13; 200mM NaOH, 1mM EDTA, 0.1% Triton X-100) at 22V for 50 min at 4°C. After the electrophoresis step, the CometChip was re-equilibrated to neutral pH using multiple washings with Tris buffer (0.4M Tris·Cl, pH7.4 and 20mM Tris·Cl, pH7.4). Subsequently, the DNA was stained with 1×SYBR Gold dye (ThermoFisher Scientific) diluted in Tris buffer (20mM Tris·Cl, pH7.4) for 30 min and de-stained for 1 hr in Tris buffer (20mM Tris·Cl, pH7.4). Next, comet images were collected using a Celigo S imaging cytometer (Nexcelom Bioscience; Lawrence, MA) at 1 micron/pixel resolution. The

comets and the indicated DNA damage were then analyzed using the dedicated comet analysis software (CAS), and the data were exported to Excel (Microsoft) and subsequently to Prism 8 (GraphPad Prism) for data presentation and statistical analysis. DNA damage is represented as % Tail DNA.

NAD^+ measurements

The NAD^+ level in extracts of each cell line was measured using the Enzychrome NAD^+/NADH colorimetric assay kit (BioAssay Systems) as we have described previously (3,10). Briefly, LN428 cells or GSCs ($3x10^5$) were seeded in a 35 mm plate in 2 mL growth medium and allowed to grow overnight. NRH (or other metabolites, as indicated) was added to a final concentration of 100μM. The cells then were collected at 0.5, 1, 2, 4, 6, or 8 hours post-treatment and the cells treated with DMSO for 8 hours was used as the normalization control. Cell pellets were homogenized using plastic pestles and the extraction of NAD^+ was performed using the extraction buffers provided. Extracts were heated at 60°C for 5 min and neutralized with the buffers provided. Samples were centrifuged, and the supernatant was immediately used for measurements of NAD^+ content using a Microplate Reader (BioTek) at 565 nm.

NAD^+ analysis for comparison of all four GSC cell lines

GSC-19, GSC-83, GSC-84, or GSC-326 cells ($1.0x10^6$) in 10ml growth medium were treated with NRH (100μM) or media for 6 hours. The cells were then pelleted by centrifugation (1200 rpm, 5 minutes) and culture media was removed. The cell pellets were then washed with 1X PBS. The cells were pelleted again by centrifugation and PBS was removed. Each cell pellet was lysed in NaOH (0.2N, 100μL) for five minutes at room temperature. The protein concentration for each sample was measured using a Nanodrop Spectrophotometer (ThermoFisher Scientific) at 280nm. The NAD^+ concentration for each cell line was then

measured using the Promega NAD^+/NADH-Glo Assay kit (cat#G9071, Promega) as per the manufacturer's instructions. The volume of each sample was diluted 1:50 in dilution buffer (equal volume of 0.2N NaOH and 1X PBS) and 50µL of each sample was added in duplicate to wells of a white, clear bottom 96-well dish. For NAD^+ measurements, HCl (0.4N, 25µL) was added to each well and the plate was incubated at 60°C for 15 minutes, followed by incubation at room temperature for 10 minutes. The samples were neutralized by adding Tris-Cl pH 10.7 (25µL) to each well. The detection reagent (100µL) was added to each well and luminescence was recorded using a luminometer. NAD^+ standards were prepared in dilution buffer (equal volume of 0.2N NaOH and 1X PBS or equal volume of 0.4N HCl and 1X PBS), as indicated in the assay protocol. The NAD^+ concentration was calculated and normalized to the protein concentration of each sample.

Poly-(ADP-ribose) ELISA

Poly-(ADP-ribose) (PAR) was measured in LN428 cells using the HT PARP *in vivo* Pharmacodynamic ELISA Kit II (R&D Systems). LN428 cells ($3x10^5$) were seeded in a 35 mm plate in growth medium (2mL) and allowed to grow overnight. The following day, cells were treated with NRH (100µM). Cells were collected at 0, 2, 4, 6 and 8 hours. Cells were trypsinized, counted and centrifuged at 250 x g for 5 minutes at 5°C. The cell pellet was resuspended in 1X PBS (1mL) and at a concentration of 1x 10^6 cells/mL in 1.5 mL microcentrifuge tubes and centrifuged at 10,000 g at 5°C for 10 seconds. Supernatant was removed and cells were lysed in Cell Lysis Buffer (500µL) included in the kit. SDS (at a final concentration of 1%) was added to each sample and cell extracts were incubated at 100°C for 5 minutes. A 0.01 volume of 100x magnesium cation and 2µL DNase I (2 Units/uL) was added to each sample and incubated at 37°C for 90 minutes. Samples were centrifuged at 10,000 x g for 10 mintues and the cell pellet

was removed. The protein concentration for each sample was measured using a DC protein assay. Reagents and working standards were prepared as specified in the kit protocol. The supernantant from each sample was diluted 100x in sample buffer provided in the kit, and 50µL from each standard and test sample was added in triplicate to appropriate wells. The wells were covered with sealing film and incubated overnight. PAR polyclonal antibody was diluted 1:250 in antibody dilutant buffer and incubated at 25°C for one hour. Wells were aspirated and washed 4 times with PBST. PAR polycolonal antibody (50µL) was added to each well, the wells were covered and allowed to incubate for 2 hours. Goat anti-rabbit IgG HRP was diluted 1:250 in antibody dilutant and allowed to incubate at 25°C for one hour. Wells were washed 4x with PBST and diluted goat anti-rabbit IgG HRP (50µL) was added to each well and incubated at 25°C for 2 hours. Wash steps were repeated and 100µL PeroxyGlow reagents A and B were prepared and added to each well and chemiluminescent readings were immediately recorded.

Immunofluorescence – LN428/LivePAR cells

LN428/LivePAR cells (1.2×10^5) were seeded in an 8-well, glass bottom chamber (ThermoScientific, cat#155409PK) with 400 µl growth media and cultured overnight. The cells were then treated with either NRH (100 µM) plus PARGi (10 µM) or control media for 4 hours. The cells were washed twice with 1X PBS and fixed for 10 minutes with 4% paraformaldehyde at room temperature. Paraformaldehyde was removed and the cells were washed 2 times with 1X PBS. The PBS was removed, then 100% methanol (ice cold) was added to each well for 7 minutes (on ice). The cells were then washed with 1X PBS three times, then the cells were permeabilized with 0.05% Triton 100-X for 10 minutes. After permeabilization, the cells were blocked in 10% goat serum for 30 minutes. The anti-PCNA, clone PC-10 antibody (Millipore, cat#MABE288) was added to each well (dilution, 1:200) in 10% goat serum, 0.01% 100-X triton and 1X PBS for 1 hour. Each well was washed three times with 1X PBS and goat anti-mouse

Alexa Fluor 568 (ThermFisher Scientific, cat#A-11031) was added 1:400 in 10% goat serum, 0.01% 100-X triton and 1X PBS for 1 hour. The cells were washed 3 times with 1X PBS, and 3 drops/mL of NucBlue stain in 1X PBS was added to each well for 15 minutes. Cells were washed three times with 1X PBS and the cells were imaged at 60x resolution by confocal microscopy using a Nikon A1r confocal microscope.

Immunofluorescence – GSC-83 cells

GSC-83 cells (4 x 10^6) were cultured in 10 ml growth medium with 4 mM thymidine (Sigma, cat#T1895-10G,) for 24 hours. The cells were then collected by a centrifugation at 1200 rpm for 5 minutes then washed with 1xPBS. Cells (2 x 10^6) were then seeded into a 100 mm dish and treated with either NRH (100μM) plus PARGi (10μM) or media for 2 hours. After treatment, the cells were collected by centrifugation at 1200 rpm for 5 minutes and re-suspended into 1 ml 4% formaldehyde in PBS for 10 min at room temperature (RT) and subsequently permeabilized by a 5 min incubation in ice-cold methanol/acetone solution (1:1). The cells were washed once with 10ml 1x PBS and blocked in blocking solution (1% BSA in 1X saponin-based permeabilization and wash reagent from the Click-iT™ EdU Alexa Fluor™ 647 Flow Cytometry Assay Kit, ThermFisher Scientific, Cat# C10419). After blocking, the cells were incubated with the primary antibody (60 min, RT) in 250μl of blocking solution (PAR mouse monoclonal antibody, 10H, 1:200 or PNCA rabbit monoclonal antibody: cat#: ab92552, Abcam, 1:200) followed by 3x washing with TBST (5 min). See also **Supplementary Table S2** for details of all antibodies used. Then, the cells were incubated with the secondary antibodies in 1%BSA/1xTBST for 60 minutes at RT (Goat anti-Rabbit-Alexa Fluor 568, ThermoFisher Scientific, cat#A-11011; Goat anti-mouse-Alexa Fluor 488, ThermoFisher Scientific, cat#: A-11001), 1:400), followed by three washes (5 min in TBST). The nucleus was stained with Hoechst 33342, 5μM (Cat#: 62249, ThermoFisher Scientifc). The wells of an 8-well-glass

bottom chamber were then coated with 45 μg/ml Cell-Tak (Cat#: 354240, Corning) in 0.1M $NaHCO_3$ solution for 20 minutes at RT followed by three washes with 1ml 1xPBS. The stained GSC-83 cells were immobilized in these pre-coated 8-well-glass bottom chambers and the cells were imaged at 60x resolution by confocal microscopy using a Nikon A1r confocal microscope.

References

1. Mao, P., Joshi, K., Li, J., Kim, S.H., Li, P., Santana-Santos, L., Luthra, S., Chandran, U.R., Benos, P.V., Smith, L. *et al.* (2013) Mesenchymal glioma stem cells are maintained by activated glycolytic metabolism involving aldehyde dehydrogenase 1A3. *Proc Natl Acad Sci U S A*, **110**, 8644-8649.
2. Fang, Q., Andrews, J., Sharma, N., Wilk, A., Clark, J., Slyskova, J., Koczor, C.A., Lans, H., Prakash, A. and Sobol, R.W. (2019) Stability and sub-cellular localization of DNA polymerase beta is regulated by interactions with NQO1 and XRCC1 in response to oxidative stress. *Nucleic Acids Res*, **47**, 6269-6286.
3. Fouquerel, E., Goellner, E.M., Yu, Z., Gagne, J.P., Barbi de Moura, M., Feinstein, T., Wheeler, D., Redpath, P., Li, J., Romero, G. *et al.* (2014) ARTD1/PARP1 negatively regulates glycolysis by inhibiting hexokinase 1 independent of NAD+ depletion. *Cell reports*, **8**, 1819-1831.
4. Fang, Q., Inanc, B., Schamus, S., Wang, X.H., Wei, L., Brown, A.R., Svilar, D., Sugrue, K.F., Goellner, E.M., Zeng, X. *et al.* (2014) HSP90 regulates DNA repair via the interaction between XRCC1 and DNA polymerase beta. *Nature communications*, **5**, 5513.
5. Hsu, P.D., Scott, D.A., Weinstein, J.A., Ran, F.A., Konermann, S., Agarwala, V., Li, Y., Fine, E.J., Wu, X., Shalem, O. *et al.* (2013) DNA targeting specificity of RNA-guided Cas9 nucleases. *Nat Biotechnol*, **31**, 827-832.
6. Slyskova, J., Sabatella, M., Ribeiro-Silva, C., Stok, C., Theil, A.F., Vermeulen, W. and Lans, H. (2018) Base and nucleotide excision repair facilitate resolution of platinum drugs-induced transcription blockage. *Nucleic Acids Res*, **46**, 9537-9549.
7. Sanjana, N.E., Shalem, O. and Zhang, F. (2014) Improved vectors and genome-wide libraries for CRISPR screening. *Nat Methods*, **11**, 783-784.
8. Shalem, O., Sanjana, N.E., Hartenian, E., Shi, X., Scott, D.A., Mikkelson, T., Heckl, D., Ebert, B.L., Root, D.E., Doench, J.G. *et al.* (2014) Genome-scale CRISPR-Cas9 knockout screening in human cells. *Science*, **343**, 84-87.
9. Sykora, P., Chiari, Y., Heaton, A., Moreno, N., Glaberman, S. and Sobol, R.W. (2018) Application of the CometChip platform to assess DNA damage in field-collected blood samples from turtles. *Environ Mol Mutagen*, **59**, 322-333.
10. Wilk, A., Hayat, F., Cunningham, R., Li, J., Garavaglia, S., Zamani, L., Ferraris, D.M., Sykora, P., Andrews, J., Clark, J. *et al.* (2020) Extracellular NAD(+) enhances PARP-dependent DNA repair capacity independently of CD73 activity. *Sci Rep*, **10**, 651.
11. Koczor, C.A., Saville, K.M., Andrews, J.F., Clark, J., Fang, Q., Li, J., Al-Rahahleh, R.Q., Ibrahim, M., Makarov, M.V., Migaud, M. *et al.* (2021) Temporal dynamics of base excision / single-strand break repair protein complex assembly and disassembly are modulated by the PARP/NAD+/SIRT6 axis. *Cell Reports*, 37, 109917-109942.

Supplemental Figures

Supplement Figure S1

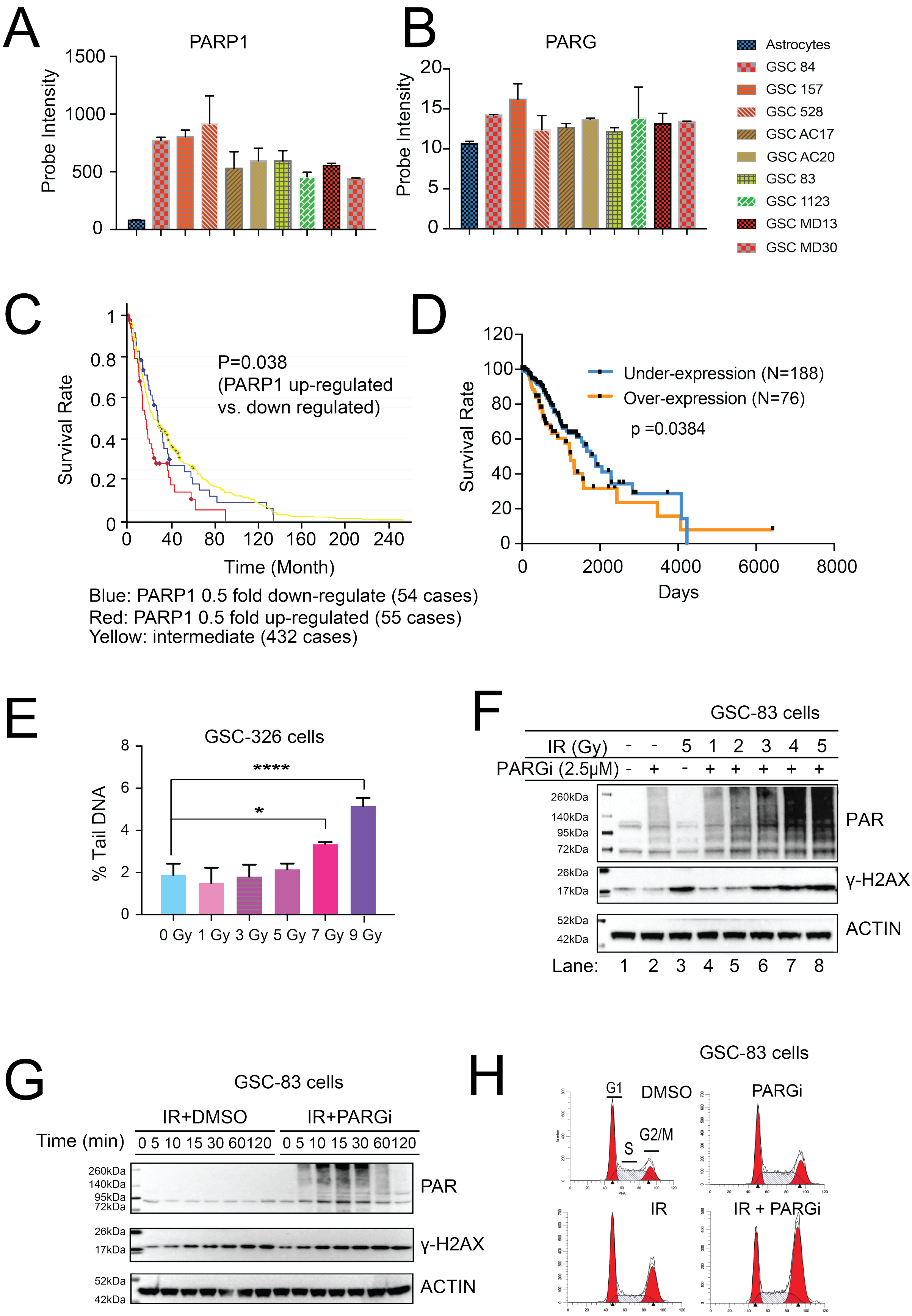

Supplement Figure S1

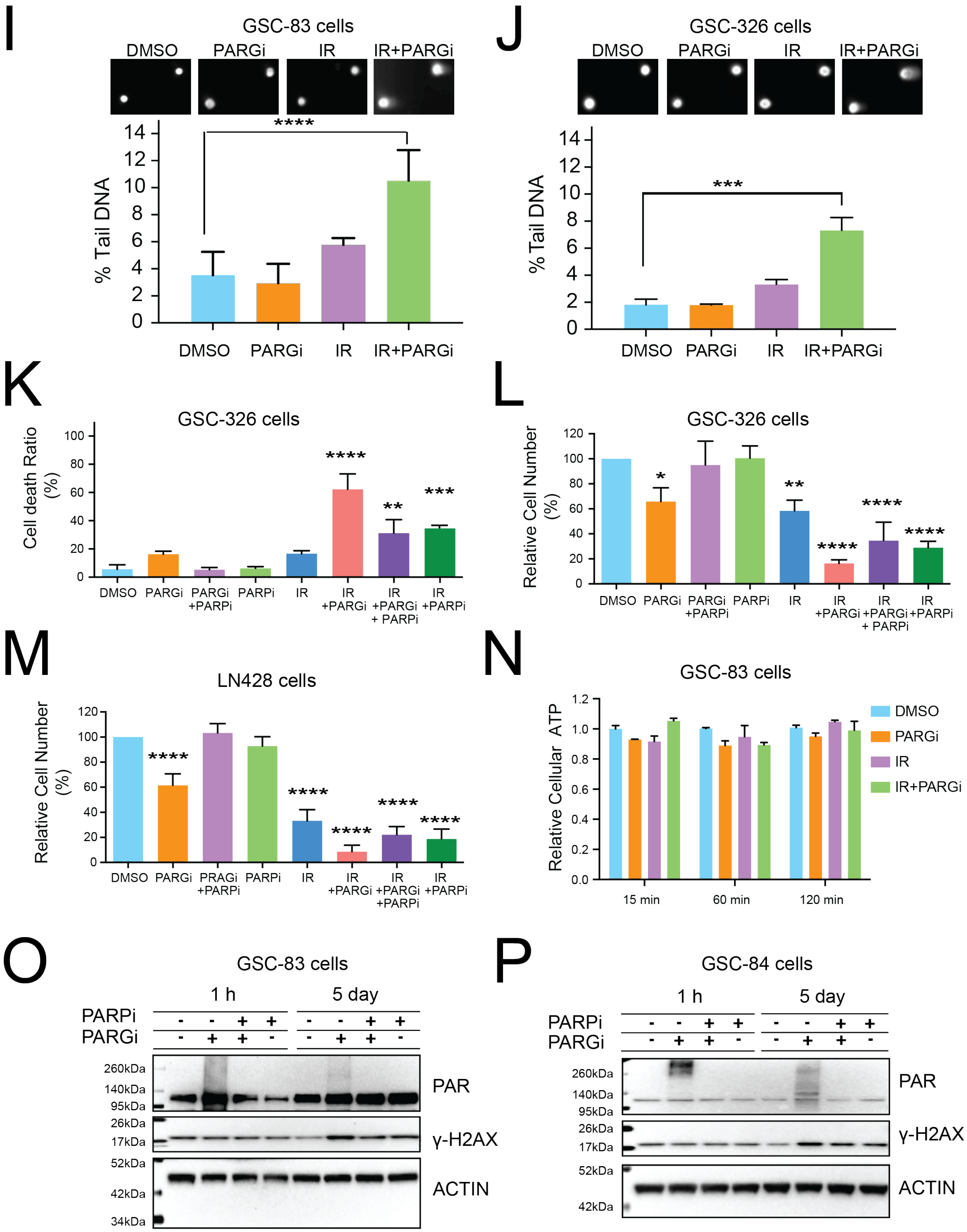

Supplement Figure S1

Q

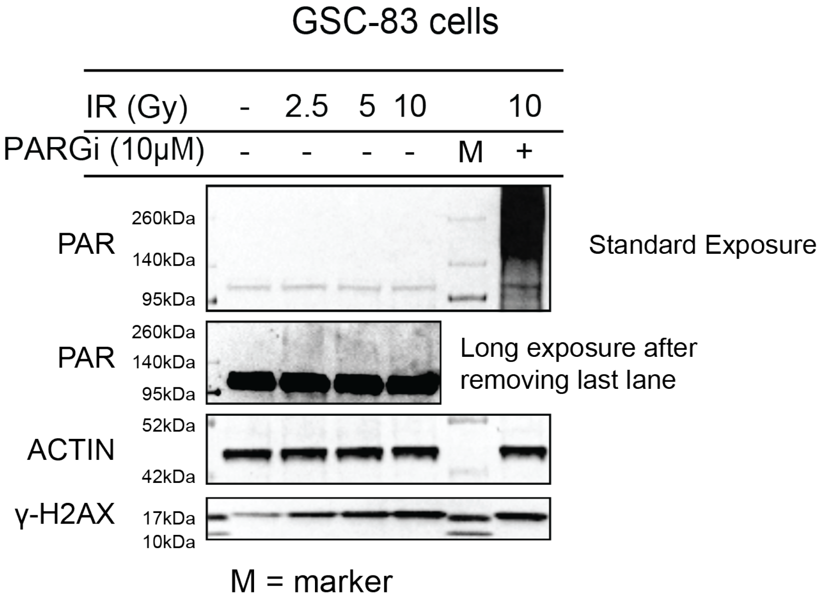

R

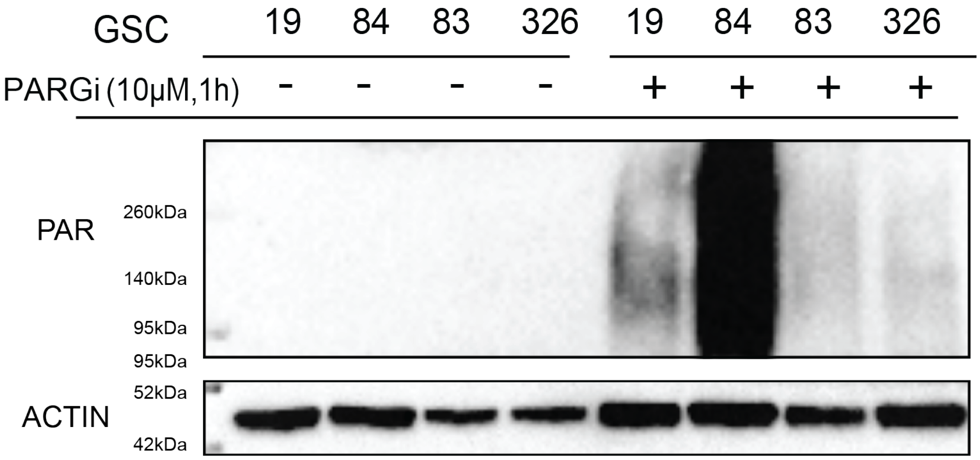

Figure S1. Up-regulated PARP1 expression in glioma stem cells – impact on PARG inhibitor response

(A) Microarray analysis comparing the mRNA levels of PARP1 in GSCs to that of normal human astrocytes.

(B) Microarray analysis comparing the mRNA levels of PARG in GSCs to that of normal human astrocytes. The key for panels A and B is shown on the right.

(C) Kaplan–Meier survival analysis comparing overall survival of 541 GBM patients shown in the REMBRANDT database according to the PARP1-based classification (Compared to the intermediate, a 0.5-fold increase in expression of PARP1 was classified as PARP1 up-regulated and a 0.5-fold decrease in expression of PARP1 was classified as PARP1 down-regulated).

(D) Kaplan–Meier survival analysis of the overall survival of 264 WHO grade III glioma patients from the TCGA database according to a PARP1-based classification.

(E) CometChip analysis of GSC-326 cells treated with IR (doses indicated). Each bar represents the mean % Tail DNA of >400 comets for each treatment, (**p*<0.05, *****p*<0.0001).

(*F*) Immunoblot analysis of PAR and γ-H2AX of whole cell lysates prepared from GSC-326 cells pre-treated with PARGi (2.5μM) for 30 minutes followed by IR treatment at the doses indicated. β-Actin was used as the loading control.

(G) Immunoblot analysis of PAR and γ-H2AX after treatment (from 5 min to 2 h) with IR (3Gy) or IR+PARGi (2.5μM). β-Actin was used as the loading control.

(H) Representative cell cycle analysis histogram of GSC-83 cells 24 hours after treatment with (DMSO), PARGi (2.5μM), IR (3Gy) or IR+PARGi.

(I) Representative CometChip images and quantification of CometChip analysis of GSC-83 cells after treatment. Each bar represents the mean % Tail DNA of 1742, 1806, 850 or 521 automatically extracted comets for each treatment: vehicle (DMSO), PARGi (10μM), IR (5Gy) or IR+PARGi grouped in 4 technical repeats, respectively (*****p*<0.0001).

(J) Representative CometChip images and quantification of CometChip analysis of GSC-326 cells after treatment: vehicle (DMSO), PARGi (10μM), IR (5Gy) or IR+PARGi, (****p*<0.001).

(K) Relative number (%) of dead cells (GCS-326 cells) 5-days after treatment: vehicle (DMSO), PARGi (10μM), PARGi+PARPi, PARPi (ABT-888,10μM), IR (5Gy), IR+PARGi, IR+PARGi+PARPi, or IR+PARPi (***p*<0.01, ****p*<0.001, *****p*<0.0001).

(L) Relative number (%) of viable GCS-326 cells, normalized to vehicle control, 5-days after treatment: vehicle (DMSO), PARGi (10μM), PARGi+PARPi, PARPi (ABT-888,10μM), IR (5Gy), IR+PARGi, IR+PARGi+PARPi, or IR+PARPi; (**p*<0.05, ***p*<0.01, *****p*<0.0001).

(M) Relative number (%) of viable LN428 cells, normalized to vehicle control, 5-days after treatment: vehicle (DMSO), PARGi (10μM), PARGi+PARPi, PARPi (ABT-888,10μM), IR (5Gy), IR+PARGi, IR+PARGi+PARPi, or IR+PARPi; (****p*<0.0001).

(N) Cellular ATP levels for GCS-83 cells at 15, 60, 120 minutes after treatment: vehicle (DMSO), PARGi, IR or IR+PARGi.

(O) Immunoblot analysis of PAR and γ-H2AX of GSC-83 cells after PARGi (10μM) treatment for 1h or 5 days. β-Actin was used as the loading control.

(P) Immunoblot analysis of PAR and γ-H2AX after 1 h and 5-day treatment of PARGi (10μM) in GSC-84 cells. β-Actin was used as the loading control.

(Q) Immunoblot analysis of PAR and γ-H2AX from GSC-83 cells treated with different doses of IR, or pre-treatment with PARGi (30 min, 10μM) followed by IR (10Gy). β-Actin was used as the loading control.

(R) Immunoblot analysis of PAR after PARGi treatment (1 hr, 10μM) in GSC-19, GSC-84, GSC-83 and GSC-326 cells. β-Actin was used as the loading control.

Supplement Figure S2

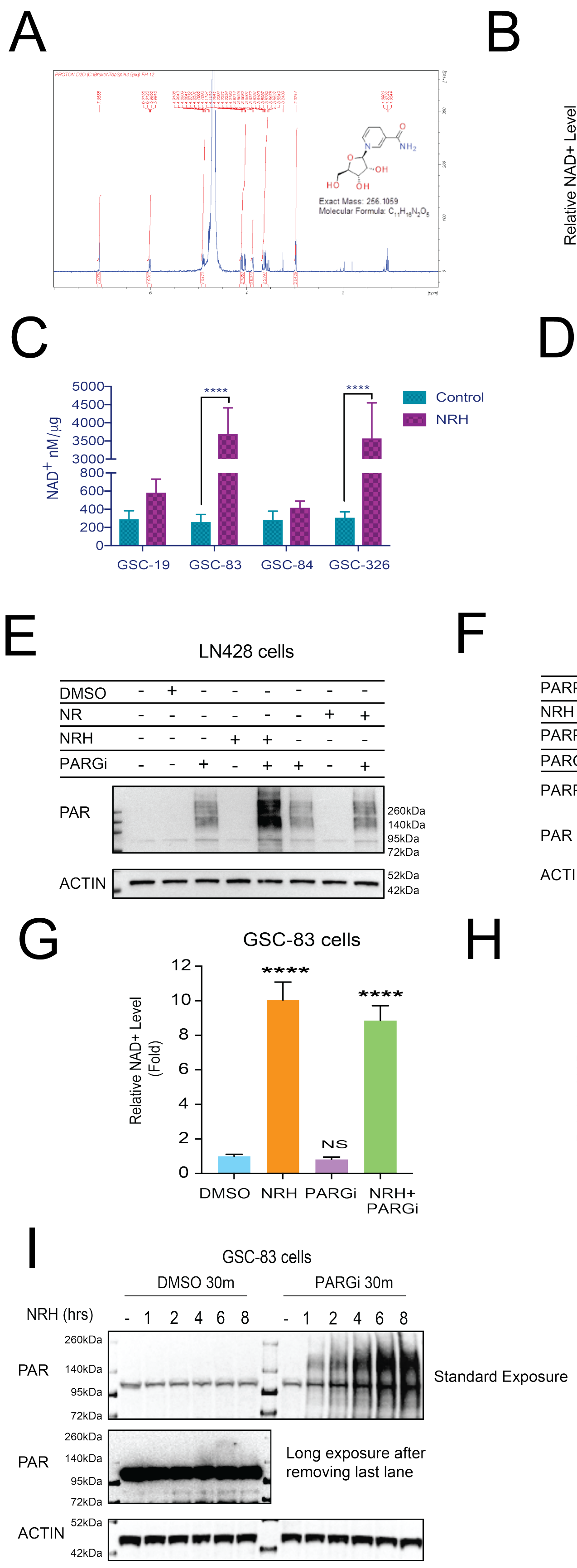

Figure S2. Dihydronicotinamide riboside (NRH) increases cellular NAD^+ levels and promotes spontaneous PAR formation to suppress replication in GSCs and glioma cells.

(A) NMR analysis showing that an aqueous solution of NRH doesn't oxidize when stored at -80ºC for two months. The chemical structure, exact mass and molecular formular are indicated on the right.

(B) Total cellular NAD^+ levels in LN428 glioma cells after treatment (4 hrs) with nicotinomide riboside (NR, 100µM), nicotinic acid riboside (NAR, 100µM), dihydronicotinamide riboside (NRH, 100µM) or dihydro-nicotinic acid Riboside (NARH, 100µM), normalized to the NAD^+ level of cells treated with vehicle (DMSO) (***p*<0.01, *****p*<0.0001).

(C) Total cellular NAD^+ levels in GSC-19, GSC-83, GSC84 and GSC-326 cells after treatment (6 hrs) with NRH (100µM) or non-treatment as the control of the basal NAD^+ level of each cell line. The NAD^+ level of each cell line was normalized to total cellular protein concentration (*****p*<0.0001).

(D) Immunoblot analysis of PARP1 and PAR in GSC-83 WT or GSC-83/PARP1-KO cells after 1 hour treatment with NRH (100µM) + PARGi (10µM). β-Actin was used as the loading control.

(E) Immunoblot analysis of PAR from total cell lysates after treatment of LN428 glioma cells with DMSO, PARGi (10µM), NRH (100µM), NRH+PARGi, NR (100µM) or NR+PARGi for 8 hrs, as indicated. β-Actin was used as the loading control.

(F) Immunoblot analysis of PARP1 and PAR from total cell lysates after treatment of LN428 or LN428/PARP1-KO glioma cells with DMSO, NRH (100µM), PARPI (10µM), PARGi (10µM), NRH+PARPi, NRH+PARGi and NRH+PARPi+PARGi, for 24 hrs, as indicated. β-Actin was used as the loading control.

(G) NAD^+ levels in GSC-83 cells after treatment with DMSO, NRH (100µM), PARGi (10µM) or NRH plus PARGi (24 hrs), normalized to the NAD^+ level of cells treated with DMSO (NS = not significant, ****p<0.0001, related to DMSO).

(H) NAD^+ levels in LN428 cells after 4 hr treatment with DMSO, NRH (100µM)+PARGi (10µM), or NRH+PARGi+5-IT (***p*<0.01).

(I) PAR (poly-ADP-ribose) immunoblot analysis of total cell lysates (GSC-83 cells) after treatment with NRH (100µM) or NRH with addition 30 minutes of PARGi (10µM) for the times indicated. β-Actin was used as the loading control.

Supplement Figure S3

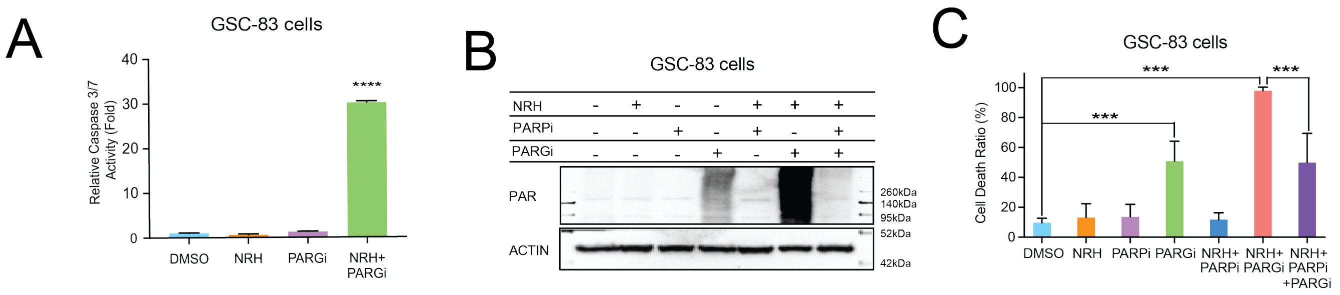

Figure S3. Selective PARGi-induced cell death in GSCs is dependent on NRH-enhanced levels of cellular NAD^+.

(A) Relative Caspase 3/7 activity of GSC-83 cells after 24h treatment with DMSO, NRH (100μM), PARGi (10μM) or NRH plus PARGi (*****p*<0.0001).

(B) Immunoblot analysis of PAR from GSC-83 cells treated (24 hours) as indicated: DMSO, NRH (100μM), PARPi (ABT-888,10μM), PARGi (10μM), NRH plus PARPi, NRH plus PARGi, NRH plus PARPi and PARGi. β-Actin was used as the loading control.

(C) Relative number (%) of dead cells (GSC-83 cells) after treatment (5 days) as indicated: DMSO, NRH (100μM), PARPi (ABT-888,10μM), PARGi (10μM), NRH plus PARPi, NRH plus PARGi, NRH plus PARPi and PARGi (****p*<0.001).

Supplement Figure S4

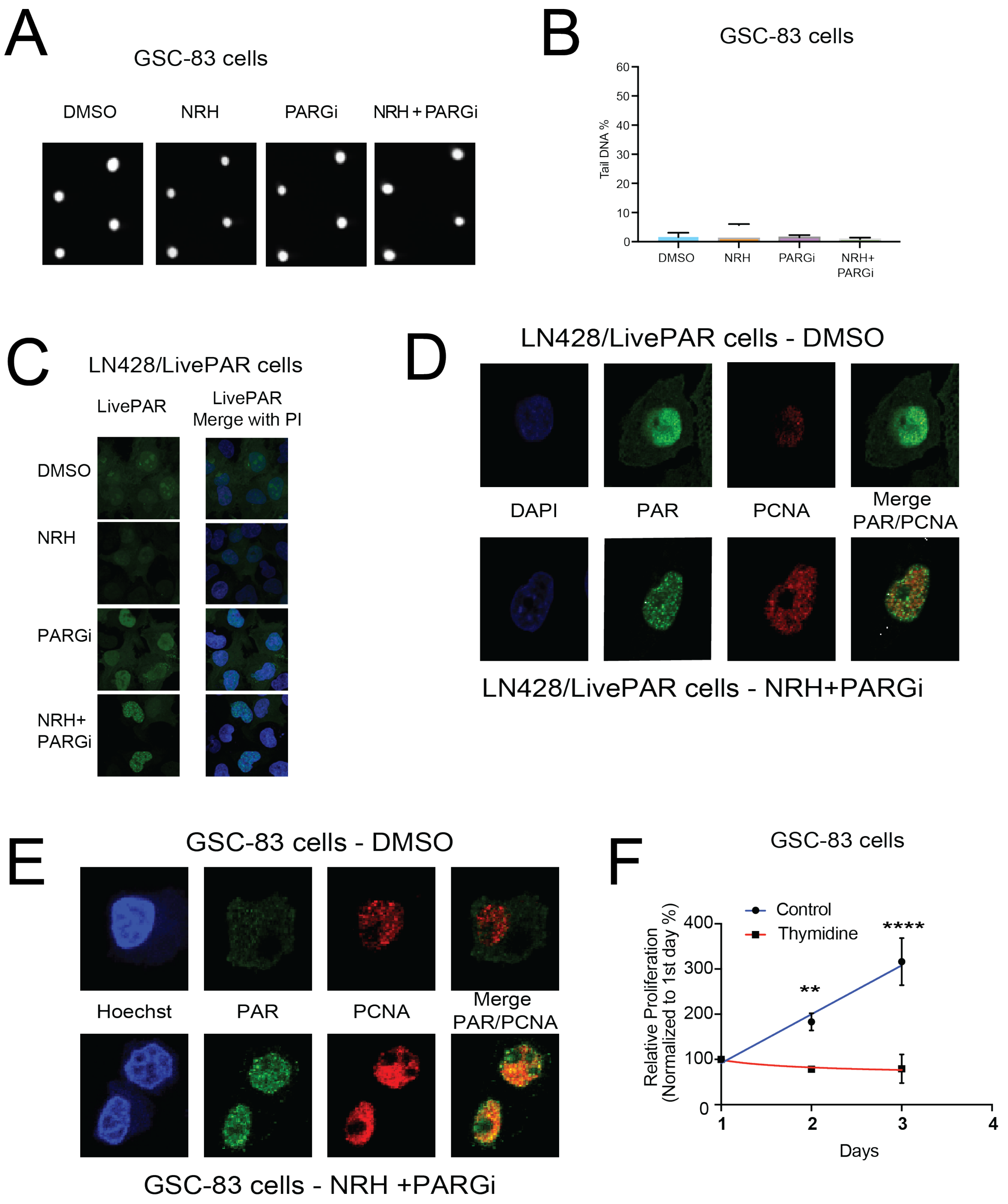

Figure S4. PARGi-induced S-phase arrest and checkpoint activation requires enhanced cellular NAD^+ from NRH exposure.

(A,B) Representative CometChip images (*A*) and quantified DNA damage analysis (*B*) revealing no detectable DNA damage in GSC-83 cells following treatment with DMSO, NRH, PARGi, or NRH plus PARGi, after 24 hours.

(*C)* Fluorescent confocal images revealing nuclear foci of PAR, as detected with the EGFP-WWE domain PAR probe (LivePAR) following expsoure of the LN428/LivePAR cells to DMSO, NRH (100μM), PARGi (10μM) or NRH plus PARGi (4 hrs).

(*D)* Fluorescent confocal images of LN428/LivePAR cells, revealing colocalization of NRH/PARGi-induced nuclear foci of PAR, as detected with the EGFP-WWE domain PAR probe (LivePAR) and of PCNA, as detected by immunofluorescence. Cells treated with DMSO (control) or NRH/PARGi, 4 hrs.

(*E)* Fluorescent confocal images of GSC-83 cells, revealing colocalization of NRH/PARGi-induced nuclear foci of PAR and of PCNA, as detected by immunofluorescence. Cells treated with DMSO (control) or NRH/PARGi, 2 hrs.

(F) Relative proliferation of GSC-83 cells after 72h treatment with 2mM thymidine (**$p<0.01$, ***$p<0.001$; related to the control).

Supplement Figure S5

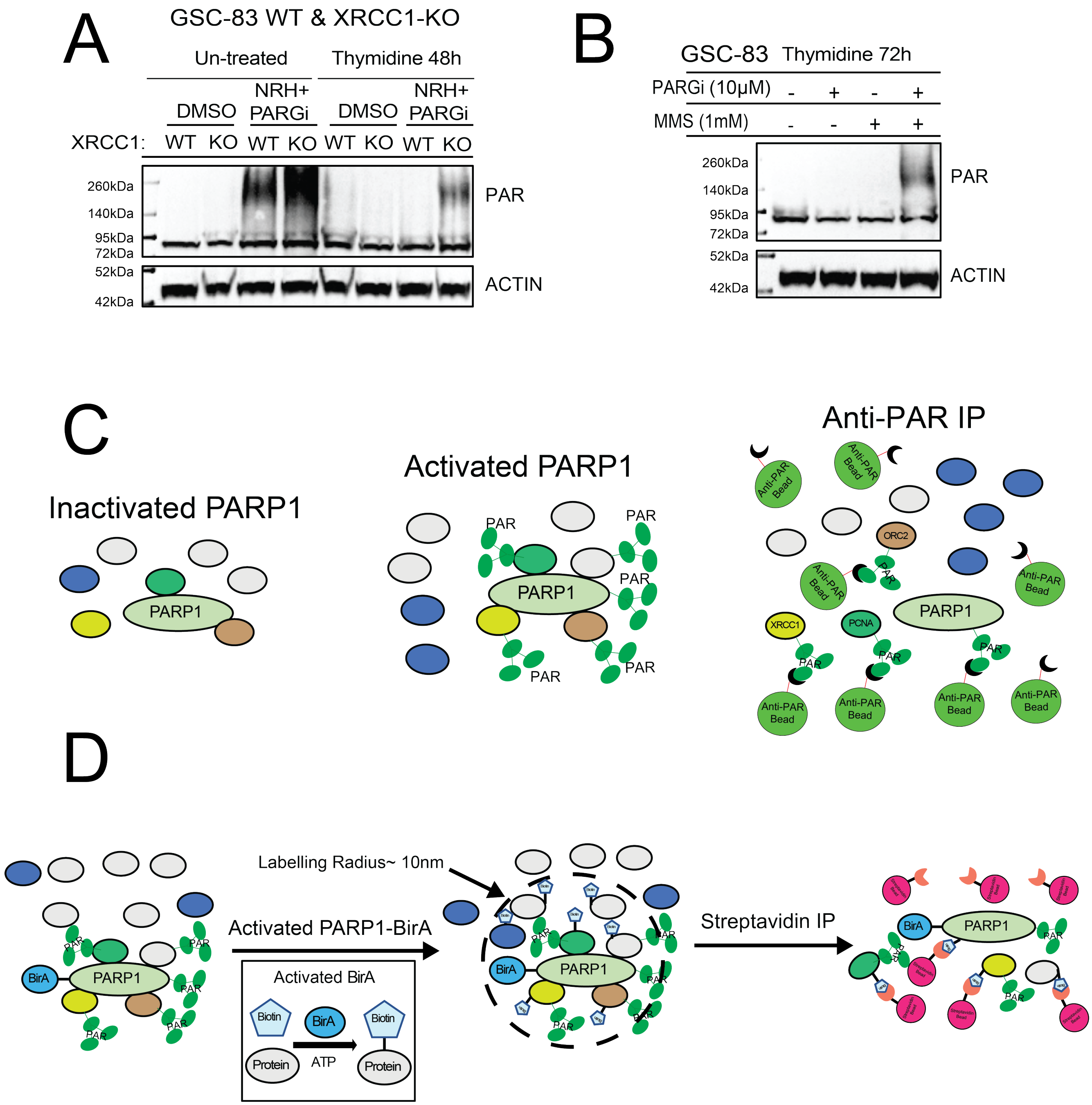

Figure S5. Replication-associated PARP1 activation coordinates BER/SSBR pathway engagement and PAR-induced assembly of replication complex proteins.

(A) Immunoblot analysis of PAR in GSC-83/WT or GSC-83/XRCC1-KO cells either untreated or treated with thymidine (2mM) for 48 hours followed by an additional treatment with DMSO, or NRH (100μM) plus PARGi (10μM) for 1 hour. β-Actin was used as the loading control.

(B) Immunoblot analysis of PAR in GSC-83 cells after 72h treatment with thymidine (2mM) followed by an additional treatment with DMSO, MMS (1mM), PARGi or MMS+PARGi for 30 minutes, as indicated. β-Actin was used as the loading control.

(C) A cartoon to illustrate PAR-IP: The activated PARP1 covalently modifies target proteins with poly-ADP-ribose (PAR). The PAR modified proteins are captured using a specific PAR binding resin, comprised of the Af1521 macrodomain bound to agarose beads.

(D) A cartoon to illustrate the PARP1/BioID system: A PARP1-biotin ligase (BirA-R118G) fusion (PARP1-BirA) protein is expressed in GSC-83 cells or LN428 cells. Upon actiavation with the addition of biotin, the PARP1-BirA covalently adds biotin to proteins among the PARP1-protein complexes, within 10nm of the PARP1-BirA fusion protein. Then, the streptavidin-conjugated beads capture the biotin modified proteins.

Supplementary Table S1. Human cell lines developed for and/or used in this study

Cell line name	Cell line description	Growth media*
GSC-83	Glioma stem cells, Mesenchymal (MES) subtype	Media #1
GSC-326	Glioma stem cells, Mesenchymal (MES) subtype	Media #1
GSC-19	Glioma stem cells, Proneural (PN) subtype	Media #1
GSC-84	Glioma stem cells, Proneural (PN) subtype	Media #1
GSC-83/PARP1-KO	GSC-83 cells with PARP1 gene KO via CRISPR/Cas9	Media #1
GSC-83/XRCC1-KO	GSC-83 cells with XRCC1 gene KO via CRISPR/Cas9	Media #1
LN428	Glioblastoma cell line	Media #2
LN428/PARP1-KO	LN428 cells with PARP1 gene KO via CRISPR/Cas9	Media #3
LN428/XRCC1-KO	LN428 cells with XRCC1 gene KO via CRISPR/Cas9	Media #3
LN428/LivePAR	LN428 cells expressing LivePAR (11)	Media #4
Astrocyte	Norman human Astrocytes (Lonza)	Media #5

*Media #1 (GSC growth Medium): DMEM-F12 (Cat# 10565, Life Technologies) supplemented with B27 (1:50), heparin (5 mg/mL), basic FGF (bFGF) (20 ng/mL), EGF (20 ng/mL) and 1x antibiotic/antimycotic (Life Technologies; #15240-096).

*Media #2: MEM-a with 10% FBS (10%, heat inactivated), 1x antibiotic/antimycotic (Life Technologies; #15240-096) and Gentamycin (5µg/mL).

*Media #3: Media #2 supplemented with Puromycin (1.0µg/ml).

*Media #4: Media #2 supplemented with Hygromycin (200µg/ml).

*Media #5: AGM TM Astrocyte Growth Medium BulletKit (Catalog #CC-3186, Lonza)

Supplementary Table S2. Primary and secondary antibodies used in this study

Target Antigen	Procedure	Company	Catalogue #	Dilution
PARP1	Immunoblot	Novus Biologicals	MAB8095	1:1000
XRCC1	Immunoblot	Bethyl	A300-065A	1:1000
γ-H2AX	Immunoblot	Cell Signaling Technology	9718s	1:1000
CHK1	Immunoblot	Cell Signaling Technology	2G1D5	1:1000
phosphorylated CHK1	Immunoblot	Cell Signaling Technology	2348S	1:1000
β-Actin	Immunoblot	Cell Signaling Technology	8457S	1:2000
PAR	Immunoblot	Gift from Mathias Ziegler (University of Bergen, Norway)	N/A	1:1000
Cleaved Caspase 3	Immunoblot	Cell Signaling Technology	9661S	1:1000
PCNA	Immunoblot	Santa Cruz Biotechnique	sc-56	1:1000
RAP (34)	Immunoblot	New Markers	9H8	1:1000
ORC2	Immunoblot	Invitrogen	PA5-67313	1:1000
Immun-Star Goat anti-mouse-HRP conjugate	Immunoblot	Bio-Rad	170-5047	1:2500
Immun-Star Goat anti-rabbit-HRP conjugate	Immunoblot	Bio-Rad	170-5046	1:2500
PCNA	Immunofluorescence	Millipore	MABE288	1:200
PCNA	Immunofluorescence	Abcam	ab92552	1:200
Mouse anti-PAR (10H)	Immunofluorescence	Generous gift from Mathias Ziegler (University of Bergen, Norway)	n/a	1:200
Goat anti-Rabbit-Alexa Fluor 568	Immunofluorescence	ThermoFisher Scientific	A-11011	1:400
Goat anti-mouse-Alexa Fluor 488	Immunofluorescence	ThermoFisher Scientific	A-11001	1:400

Supplementary Table S3. Vectors developed for and used in this study

Source	Plasmid name	Insert description	gRNA sequence
Kindly provided by Wim Vermeulen, Erasmus MC, Netherlands (6)	pLentiCRISPRv2-PARP1-exon4	Cas9 plus PARP1 gRNA exon 4; contains a puromycin resistance cassette	GGTCCACCATCTTCTTGGAC
Kindly provided by Wim Vermeulen, Erasmus MC, Netherlands (6)	pLentiCRISPRv2-XRCC1-exon3	Cas9 plus XRCC1 gRNA exon 3; contains a puromycin resistance cassette	AGTGTGGACATTGGGAATGA
(11) (available at Addgene #176063)	pLV-EF1A-LivePAR-Hygro	EGFP fused to the C-terminus of a WWE domain & a hygromycin resistance cassette	N/A
